# Supplementary material for: Planarian progenitor responses to injury are not specific to missing tissue identity
Source: Development. 2026 Oct 15;153(20):dev205573. doi: 10.1242/dev.205573 (PMC13723425; doi:10.1242/dev.205573)
Supplement: Supplementary file 1 [file develop-153-205573-s1.pdf]

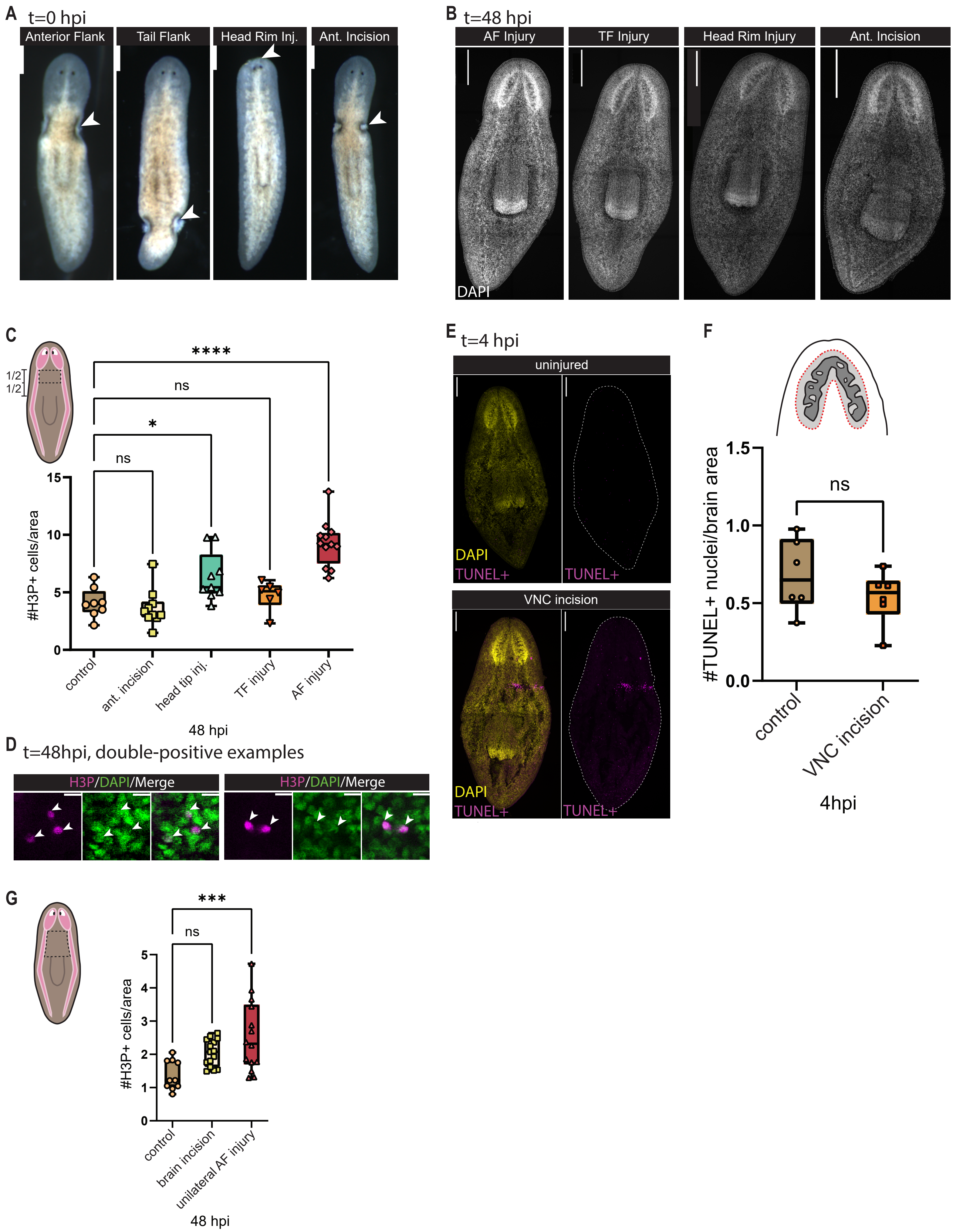

Development • Supplementary information

Fig. S1. Injury-induced proliferation and cell death responses

(**A**) Live images of injured animals immediately after injury; white arrowheads point to injury location. 200µm scale bar. (**B**) 20x confocal DAPI images representative of each injury, 48hpi. 300µm scale bar. (**C**) Number of H3P+ cells found in the prepharyngeal region as represented by the black dotted box in each injury condition. Counted with computer-aided, semi-automated methods, counting area defined by ventral nerve cords laterally and as half the distance from base of brain to top of pharynx vertically (one-way ANOVA with Dunnett's Test correction, $n \geq 6$). (**D**) Representative examples of H3P+ DAPI cells counted as H3P+, 20x, 5µm scale bar.(**E**) Representative examples of TUNEL+ staining in control and injured animals, 20x, 200µm scale bar. (**F**) Number of TUNEL+ nuclei in brain as represented in E (unpaired two-tailed Student's *t*-test, $n \geq 6$). (**G**) Number of H3P+ cells found in the prepharyngeal region as represented by the black dotted box. Counted manually, counting area defined by ventral nerve cords laterally, bottom of brain anteriorly, and top of pharynx posteriorly (one-way ANOVA with Dunnett's test correction, $n \geq 10$). (**C, F, G**) Symbols: individual animals, anonymized for scoring. Boxes represent IQR (25th-75th percentile), horizontal lines represent median, whiskers represent min. and max. values. *$p<0.05$, ***$p<0.001$, ****$p<0.0001$, n.s. = $p>0.05$. Complete n-numbers in Table S1.

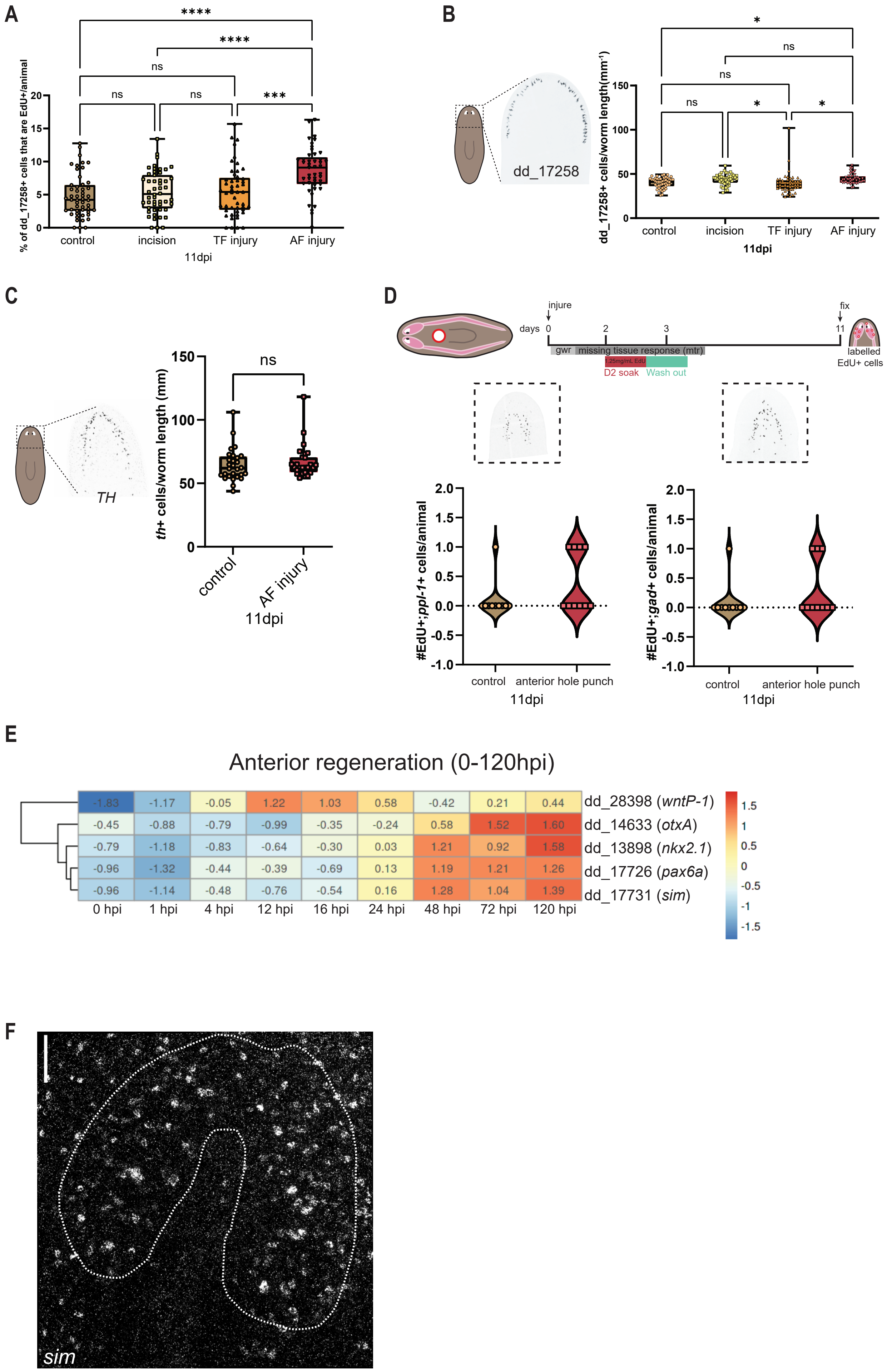
A
% of dd_17258+ cells that are EdU+/animal
control
incision
TF injury
AF injury
11dpi
****
****
ns
ns
ns
***
B
dd_17258
dd_17258+ cells/worm length(mm⁻¹)
control
incision
TF injury
AF injury
11dpi
C
TH
th+ cells/worm length (mm)
ns
control
AF injury
11dpi
D
injure
fix
days
gwr
missing tissue response (mtr)
D2 soak
Wash out
labelled EdU+ cells
#EdU+;ppl-1+ cells/animal
#EdU+;gad+ cells/animal
control
anterior hole punch
11dpi
E
Anterior regeneration (0-120hpi)
-1.83 -1.17 -0.05 1.22 1.03 0.58 -0.42 0.21 0.44 dd_28398 (wntP-1)
-0.45 -0.88 -0.79 -0.99 -0.35 -0.24 0.58 1.52 1.60 dd_14633 (otxA)
-0.79 -1.18 -0.83 -0.64 -0.30 0.03 1.21 0.92 1.58 dd_13898 (nkx2.1)
-0.96 -1.32 -0.44 -0.39 -0.69 0.13 1.19 1.21 1.26 dd_17726 (pax6a)
-0.96 -1.14 -0.48 -0.76 -0.54 0.16 1.28 1.04 1.39 dd_17731 (sim)
0 hpi 1 hpi 4 hpi 12 hpi 16 hpi 24 hpi 48 hpi 72 hpi 120 hpi
1.5
1
0.5
0
-0.5
-1
-1.5
F
sim

Fig. S2. New neuron incorporation and progenitor responses following injury outside of the brain

(A) Computer-aided, semi-automated counting of total dd_17258+ cells per animal in each injury condition, a modest increase compared to control is seen only in the AF injury condition. Statistical significance assessed by one-way ANOVA with Tukey-Kramer test correction, n≥45. **(B)** Total EdU+; dd_17258+ cells as a percentage of total dd_17258+ cells in each animal (one-way ANOVA with Tukey-Kramer test correction, n≥45). (**C**) Total *th*+ cells per animal in control and AF injury conditions (unpaired two-tailed Student's *t*-test, n≥10). (**D**) (Top) schematic: EdU delivery relative to injury and fixation and representation of hole-punch injury, (bottom) *probe*+; EdU+ cells (unpaired two-tailed Student's *t*-test, n≥28). (**E**) Heatmap showing activation of wound-induced gene expression 0-120 hpi in anterior-facing wounds of prepharyngeal region fragments. Only *wntP-1*, known to be wound-induced, shows increased activation before 48 hpi – all neural progenitor markers utilized in this study do not; data and visualization from Wurtzel et al., 2015. (**F**) Z-projected 63x confocal image of sim expression in brain, cephalic ganglia outlined with dotted white line. (**A-D**) Symbols represent individual animals, anonymized for scoring. Boxes represent IQR (25th-75th percentile), horizontal lines represent median, whiskers represent min. and max. values. *p<0.05, ***p<0.001, ****p<0.0001, n.s. = p>0.05. Complete n-numbers in Table S1.

Development • Supplementary information

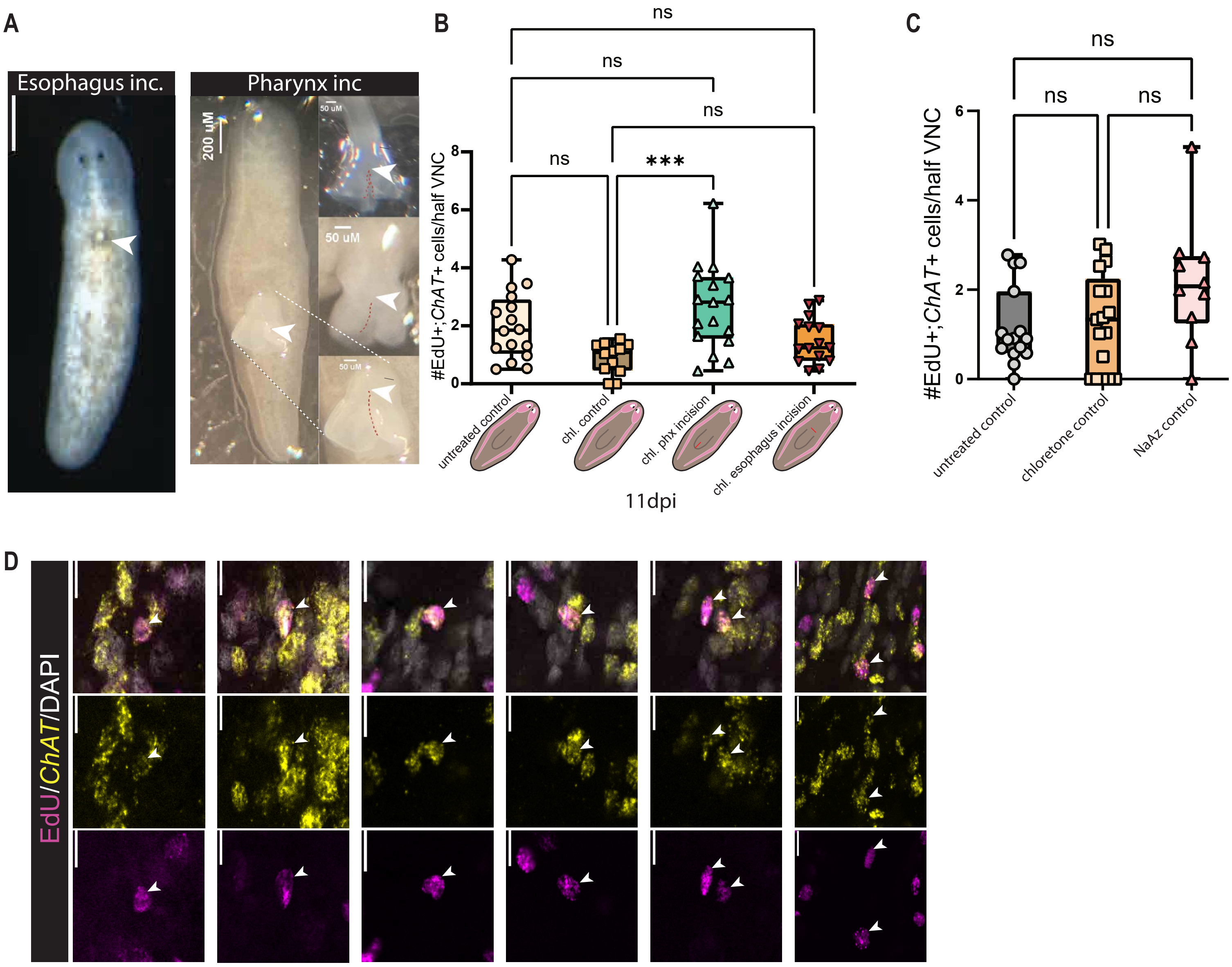

Fig. S3. New ventral nerve cord *ChAT*+ neuron incorporation following diverse injuries

(**A**) Live images of injured animals immediately after injury, white arrowheads point to injury location. Zoom in view of pharynx in pharynx incision condition, with two additional examples; dashed red line indicates incision injury edge. 200µm scale bar. (**B**) Number of EdU+; *ChAT*+ cells in each injury condition (one-way ANOVA with Tukey-Kramer test correction, n≥12). (**C**) Number of EdU+; *ChAT*+ cells in each uninjured chemical treatment condition, n≥10 (**D**) Representative images of *ChAT*+; EdU+ cells (arrowheads) counted in Figure 3. Each field of view grouped vertically. (**B-C**) Symbols represent individual animals, anonymized for scoring. Boxes represent IQR (25th-75th percentile), horizontal lines represent median, whiskers represent min. and max. values. ***$p<0.001$, n.s. = $p>0.05$. Complete n-numbers in Table S1.

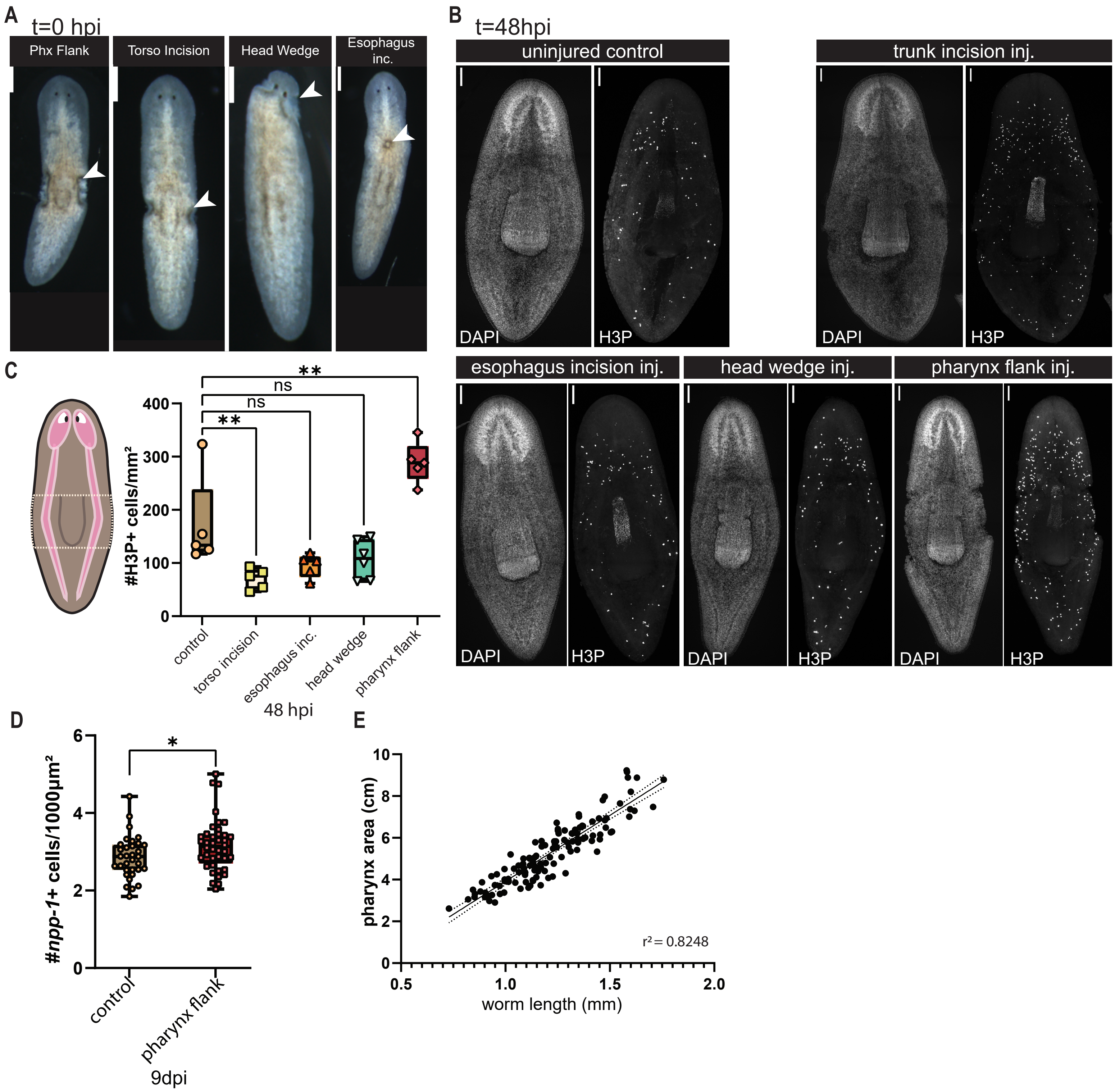

Fig. S4. Proliferative responses to injuries proximal to the pharynx

(**A**) Live images of injured animals immediately after injury, white arrowheads point to injury location. 200µm scale bar. (**B**) 20x confocal DAPI and H3P image representatives of each injury, 48hpi. 100µm scale bar. (**C**) Number of H3P+ cells in trunk region as defined by the white dotted box, H3P increase observed only for pharynx flank injury. One-way ANOVA with Dunnett's Test correction, n≥5.(**D**) Computer-aided, semi-automated counting of total *npp-1+* cells per animal in each injury condition (unpaired two-tailed Student's *t*-test, n≥19). (**E**) Simple linear regression analysis of pharynx area versus fixed worm size, r^2 = 0.8249. (**C-D**) Symbols represent individual animals, anonymized for scoring. Boxes represent IQR (25th-75th percentile), horizontal lines represent median, whiskers represent min. and max. values. *p<0.05, **p<0.01, n.s. = p>0.05. Complete n-numbers in Table S1.

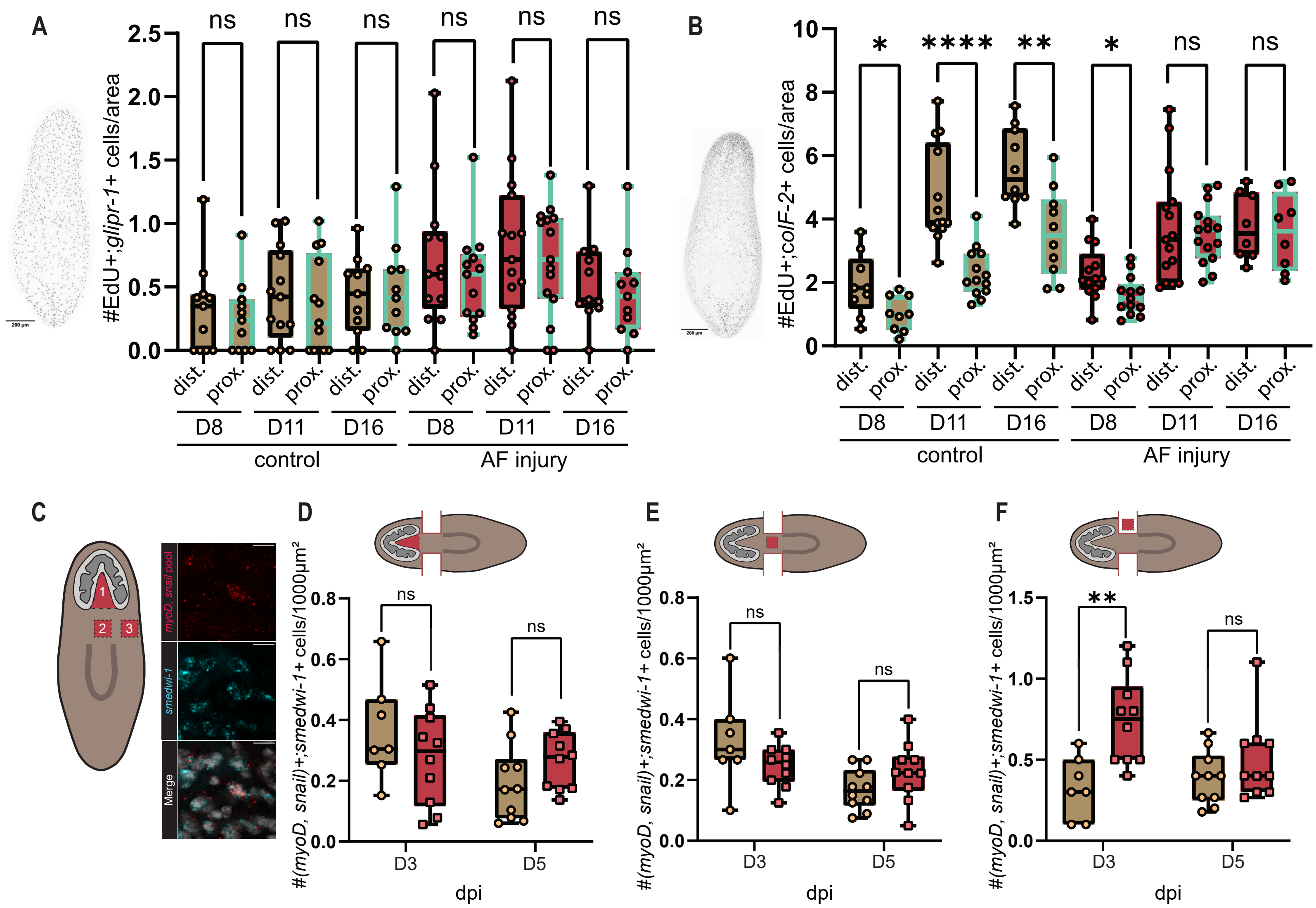

Fig. S5. Wound-proximal and wound-distal responses to injury of peripheral neurons and muscle

(**A**) Number of EdU+; *glipr-1*+ cells in wound-distal and wound-proximal areas per condition. Wound-distal is outlined in black, wound-proximal in cyan. Unpaired two-tailed Student's *t*-test per condition, n≥11. (**B**) Number of EdU+; *colF-2*+ cells in wound-distal and wound-proximal areas per condition (unpaired two-tailed Student's *t*-test, n≥8). (**C**) Counting area schematic for D-F. *myoD+, snail+;smedwi-1+* cells. *myoD+, snail+;smedwi-1+* cells in distal area (n≥7) (**D**), proximal area (n≥7) (**E**), and in-blastema (n≥7) (**F**). (**A-B, D-F**) Symbols represent individual animals, anonymized for scoring. Boxes represent IQR (25th-75th percentile), horizontal lines represent median, whiskers represent min. and max. values. *p<0.05, **p<0.01, ****p<0.0001, n.s. = p>0.05. Complete n-numbers in Table S1.

Development • Supplementary information

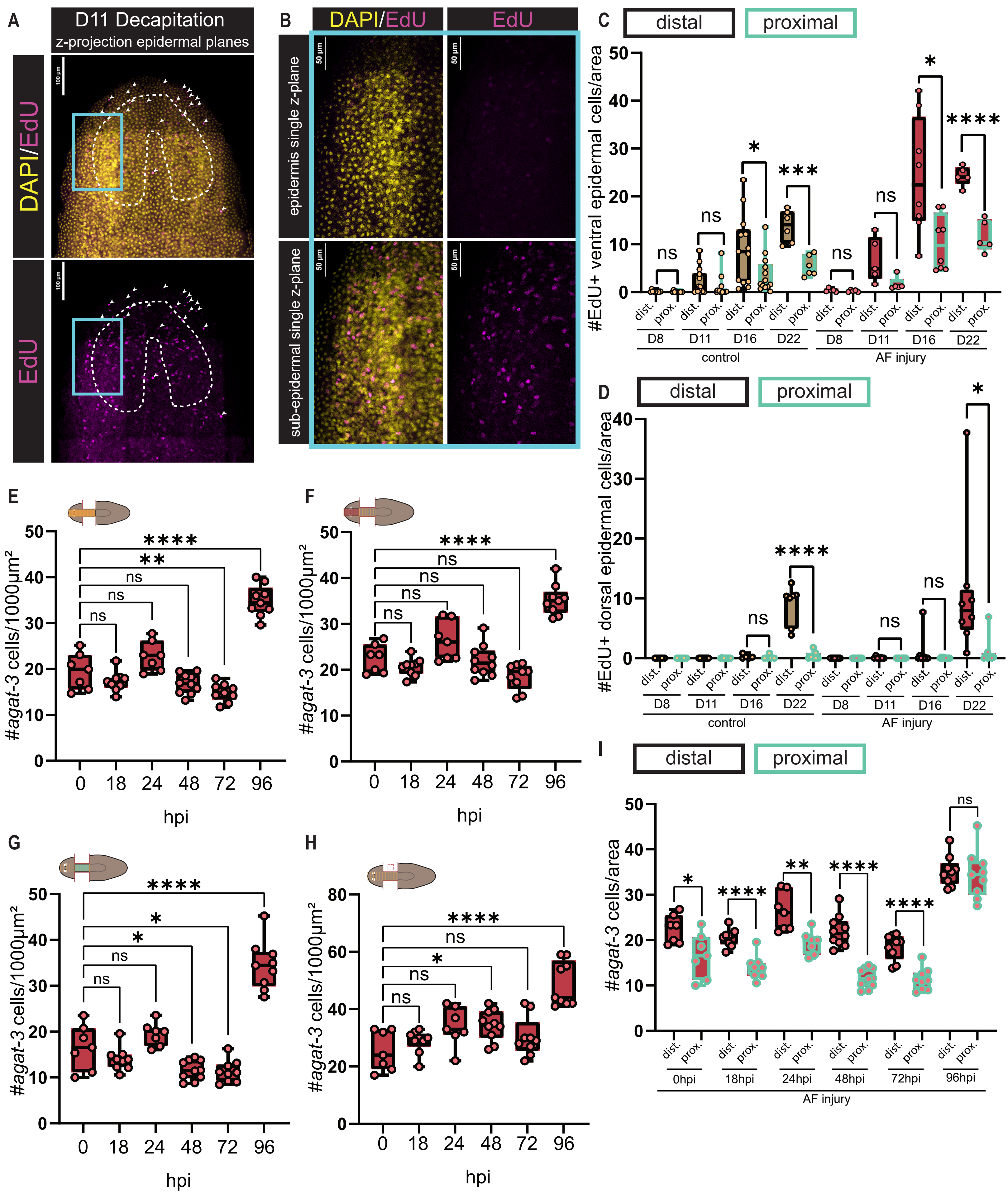

Development • Supplementary information

Fig. S6. New mature epidermal cell incorporation and post-mitotic progenitor responses in wound-proximal and wound-distal regions

(**A**) The 40x single z-plane of epidermis in 11dpi decapitation animal shown in Fig. 6B. Bleed-through of signal from subepidermal DAPI is due to curvature of animal when imaging. 100μm scale bar. Blue rectangle indicates area of interest for B. (**B**) Top: zoomed in view of epidermal z-plane. Subepidermal plane of the same animal, showing the subepidermal DAPI that bleeds through into the epidermis plane. 50μm scale bars. (**C**) Number of EdU+ ventral epidermis cells in wound-distal and wound-proximal areas per condition. Black outlines indicate wound-distal, cyan indicate wound-proximal (unpaired two-tailed Student's *t*-test, n≥5). (**D**) Number of EdU+ dorsal epidermis cells in wound-distal and wound-proximal areas per condition (unpaired two-tailed Student's *t*-test, n≥7). (**E**) Computer-aided quantification of *agat-3+* progenitors in whole area, in distal area (**F**), in proximal area (**G**), and in-blastema (**H**), (one-way ANOVA with Dunnett's test correction). (**I**) Number of *agat-3+* cells in wound-distal and wound-proximal areas per condition. Black outlines indicate wound-distal, cyan indicate wound-proximal (unpaired two-tailed Student's *t*-test). (**E-I**) n≥7. (**C-I**) Symbols represent individual animal, anonymized for scoring. Boxes represent IQR (25th-75th percentile), horizontal lines represent median, whiskers represent min. and max. values. *p<0.05, **p<0.01, ***p<0.001, ****p<0.0001, n.s. = p>0.05. Complete n-numbers in Table S1.

Table S1. Numbers for all conditions reported

A list of n-numbers for all conditions in every figure panel.

Available for download at
https://journals.biologists.com/dev/article-lookup/doi/10.1242/dev.205573#supplementary-data

Table S2. Summary table of key details for each injury and experimental paradigm

Summary table including: the injury, whether it removed tissue, if it causes a generic wound response, if it causes a missing tissue response, time of fixation/analysis, EdU labeling relative to injury, length of EdU labeling, target tissue, proximity of injury to target tissue, and regenerative outcome.

Available for download at
https://journals.biologists.com/dev/article-lookup/doi/10.1242/dev.205573#supplementary-data
