# Supplementary material for: Planarian progenitor responses to injury are not specific to missing tissue identity
Source: Development. 2026 Oct 15;153(20):dev205573. doi: 10.1242/dev.205573 (PMC13723425; doi:10.1242/dev.205573)
Supplement: Supplementary file 2 [file develop-153-205573_review_history.pdf]

Planarian progenitor responses to injury are not specific to missing tissue identity

Cecilia Pellegrini and Peter W. Reddien
DOI: 10.1242/dev.205573

Editor: Kenneth Poss

Review timeline

Original submission:	10 February 2026
Editorial decision:	21 March 2026
First revision received:	15 July 2026
Editorial decision:	18 August 2026
Second revision received:	28 August 2026
Accepted:	2 September 2026

Original submission

First decision letter

MS ID#: dev.205573

MS TITLE: Lack of specificity of progenitor responses to injury in regeneration

AUTHORS: Cecilia Pellegrini; Peter W Reddien

Dear Peter,

I have now received all the referees' reports on the above manuscript, and have reached a decision. The referees' comments are appended below.

As you will see, the referees express considerable interest in your work, but have some significant criticisms and recommend a substantial revision of your manuscript before we can consider publication. If you are able to revise the manuscript along the lines suggested, which may involve further experiments, I will be happy receive a revised version of the manuscript. Your revised paper will be re-reviewed by one or more of the original referees, and acceptance of your manuscript will depend on your addressing satisfactorily the reviewers' major concerns. Please also note that Development will normally permit only one round of major revision. If it would be helpful, you are welcome to contact us to discuss your revision in greater detail. Please send us a point-by-point response indicating your plans for addressing the referees' comments, and we will look over this and provide further guidance.

Please attend to all of the reviewers' comments and ensure that you upload both a 'clean' version of your Word file, along with a highlighted version clearly showing where you have made changes in the revised manuscript. Please avoid using 'Tracked changes' in Word files as these are lost in PDF conversion. I should be grateful if you would also provide a point-by-point response detailing how you have dealt with the points raised by the reviewers in the 'Response to Reviewers' box. If you do not agree with any of their criticisms or suggestions please explain clearly why this is so.

Reviewer 1

SUMMARY OF THE ADVANCE MADE IN THIS PAPER AND ITS POTENTIAL SIGNIFICANCE TO THE FIELD

© 2026. Published by The Company of Biologists under the terms of the Creative Commons Attribution License (https://creativecommons.org/licenses/by/4.0/).

Pellegrini and Reddien addresses an important question in regeneration biology: how regenerative cell production is tailored to missing tissues. Using planarians and a set of carefully chosen "proximal but not ablative" injuries, the authors argue that injury identity has limited influence on progenitor output and that increased proliferation within broad fate-specification zones is sufficient to drive increased incorporation into multiple tissues, including uninjured ones ("bystander effect"). The work is conceptually clear and experimentally broad.

SUGGESTIONS TO AUTHORS
Major Comments
The two main points that, in my view, would most strengthen the paper are: (i) sharper separation of "incorporation" vs "net cell number / functional replacement" across tissues and time, (ii) additional controls/analyses that reduce alternative interpretations tied to axonal injury and systemic effects (apoptosis, remodeling).

1. "Incorporation" is not equivalent to "regenerative need" or "specific replacement"
Much of the evidence is based on EdU incorporation into marker-positive mature cells (and some progenitor markers), interpreted as "amplified production." This is valid for showing changes in lineage flux, but it does not always establish whether the tissue is being overproduced, maintained, or remodeled.

For each tissue class, explicitly state whether the claim is increased flux (EdU+ fraction/number) and/or increased mature cell number.
Where feasible, add or expand analyses of total cell numbers (or density) over time in the same structures in which incorporation is quantified (brain neuron subtypes, VNC cholinergic neurons, pharynx markers). The authors already provide limited total counts for dd_17258+ (supplement), but the interpretation remains cautious.

2. Alternative interpretation: subtle injury to projections or local microdamage
The manuscript addresses this concern in multiple places (e.g., head tip injury for brain; esophagus incision for VNC), which is good. However, because the core claim challenges surveillance -like models, the standard for excluding "unnoticed damage" should be high.

For the brain bystander effect: consider adding an assay for axon integrity (e.g., pan-neuronal labeling of tracts) after each injury type used to argue "no brain damage," or include confocal views demonstrating that major tracts are intact.
For pharynx removal and VNC incorporation: given that VNC bisection did not increase new ChAT+ cells but pharynx removal did, it would help to quantify proliferation spatial maps (or at least regional H3P) in the relevant segment for each condition to directly connect "proliferation near specification zones" to the effect size.

Minor Comments
1. The epidermis section is one of the clearest arguments: blastema epidermis is derived largely from pre-existing post-mitotic progenitors; injury-induced epidermal amplification arrives later and broadly. This is convincing as presented, but it would help to connect these results more directly to the paper's main "specificity" thesis. However, make explicit whether epidermis represents an "exception that proves the rule" (i.e., turnover pipeline dominates acute repair) or a qualitatively different regulatory strategy.

2. Define "bystander effect," "target-blind," "specificity," and "surveillance-like" early and use them consistently, ideally with a simple schematic that maps each to measurable outcomes. (Please note that bystander effect is also used in cell-ablation system).

AI Usage Declaration:
Reviewer's response in bullet points were uploaded to ChatGPT5.2 to improve grammar and text. No part of manuscript or abstract was uploaded.

Reviewer 2

SUMMARY OF THE ADVANCE MADE IN THIS PAPER AND ITS POTENTIAL SIGNIFICANCE TO THE FIELD

© 2026. Published by The Company of Biologists under the terms of the Creative Commons Attribution License (https://creativecommons.org/licenses/by/4.0/).

This manuscript by Pellegrini et al. addresses an important question in planarian regeneration: whether or not stem cell responses are specific to the identity of missing tissues after injury. Using EdU incorporation together with mature cell type markers, the authors examine multiple injury paradigms across several tissue systems and show that proximal injuries that spare mature tissues can nevertheless increase progenitor incorporation into spatially restricted neuronal populations. These data provide interesting insight into regenerative dynamics and suggest a bystander like mode of incorporation, and in many respects are conceptually consistent with the authors' earlier work (e.g. Wurtzel et al., 2015; Atabay et al., 2018).

SUGGESTIONS TO AUTHORS

However, I believe there are several conceptual issues that need to be addressed before the proposed model can be generalized and the conclusions fully supported. In particular, the choice and interpretation of markers for neural progenitors require more careful justification, and prior observations and models in the field that bear directly on this question are not sufficiently considered. Overall, I think the data should be interpreted more cautiously, and the manuscript would benefit from a clearer integration with existing literature.

Major points:

1. Line 160: The authors define "neural progenitors" as piwi1^{+}/pax6a^{+} cells. It is unclear whether this population corresponds to a clearly defined neural progenitor subset or broadly labels heterogeneous cell types. Pax6a is not a specific neural progenitor marker in planarians. The authors should include additional and more specific neural progenitor markers (e.g. soxB family members, hesl3) and, where possible, markers for defined neural progenitor subtypes (e.g. Wyss et al., 2023) to substantiate their claims.
2. The authors propose an early, cross tissue "generic response" that is non specific with respect to tissue identity. If the early response is indeed an imprecise, progenitor level reaction, one would expect to detect a concomitant increase in other lineage progenitors in the same regions, not only in neurons. It is therefore unclear why comparable quantitative analyses were not performed for other lineages. Assessing proliferation of muscle, pharyngeal, intestinal, and epidermal progenitors using established lineage markers would be critical to determine whether these progenitor pools exhibit similar bystander like amplification, or whether the effect is in fact biased toward the neural lineage.
3. The manuscript overlooks the heterogeneity of neoblast and progenitor populations, particularly tgs1+ neoblasts and other progenitors, which have been shown to both respond to amputation (Zeng et al., 2018) and may contain neural progenitors (Raz et al., 2021). The authors should clarify how tgs1+ neoblasts behave during regeneration and whether the progenitor response is a general neoblast injury induced reaction or reflects a more specialized response from the regenerating sensory nervous system. The possibility that injury responsive tgs1+ neoblasts or sensory neurons may serve as part of a surveillance mechanism should be explicitly considered and addressed in the manuscript.
4. In addition, post mitotic injury induced cell states that arise in response to damage was observed in Benham Pyle et al. (2021). Tgs1 staining showed a significant increase in pluripotent neoblasts after injury (Zeng et al., 2018). The pax6a^{+} cells quantified here could, at least in part, correspond to such injury induced states rather than bona fide neural progenitors. The manuscript should consider and explicitly discuss this possibility, and clarify how the proposed model of non specific progenitor responses is reconciled with existing models involving post mitotic injury induced cell states and pluripotent neoblast expansion.
5. Several key claims rely on bulk quantification of EdU^{+} cells as evidence for generic amplification. This metric alone cannot distinguish lineage specific from lineage agnostic responses. A more informative approach would be to quantify multiple lineage specific progenitor markers in parallel within key "bystander" regions (e.g. dd_554^{+} zones) at early post injury time points. Demonstrating coordinated amplification across unrelated progenitor pools, rather than in a single lineage, would provide much stronger support for a generic response model. Moreover, increased incorporation of EdU^{+} cells does not necessarily indicate that progenitor specification is non specific. The final count of EdU^{+} mature cells may reflect various processes, including proliferation, fate specification, local retention, survival, and differential integration. The authors should at least discuss these caveats.

© 2026. Published by The Company of Biologists under the terms of the Creative Commons Attribution License (https://creativecommons.org/licenses/by/4.0/).

6. Line 116: The head tip injury may also affect mechanosensory neurons (Ross et al., 2018), potentially influencing the VNCs. Therefore, the data do not fully rule out the possibility that the observed bystander effect in the brain could result from surveillance based detection of damage by sensory neurons. This possibility should be explicitly discussed, and the authors should clarify to what extent their data distinguish between a generic cross tissue response and a sensory neuron-mediated surveillance mechanism.
7. The manuscript's central conclusion seems overstated relative to the data presented and the broader context of the field. While the study demonstrates broad injury-associated amplification and bystander-like incorporation, the title and several summary statements (e.g., lines 538-542) overstate the claim that the identity of the missing tissue has minimal impact on determining stem cell response specificity. This assertion should be moderated. Targeted regeneration studies support a more nuanced perspective that acknowledges both "specific" and "non-specific" regeneration (Bohr et al., 2021; Vu et al., 2015). A more defensible framing would emphasize that generic amplification and bystander incorporation are important components of regeneration, without dismissing identity based specificity. The title, abstract, and discussion should be revised accordingly and explicitly limited to planarians.
8. It appears that the authors interpret the results of "increased EdU incorporation into mature cell types" as broader statements regarding "nonspecific stem cell/progenitor responses." If this is the case, why not directly quantify muscle progenitors using established markers (e.g., MyoD) in figures such as Figures 5A and G-J? The same reasoning applies to Figures 6C-J, where co-staining and direct counts of epidermal progenitor populations would offer further clarity. For enhanced rigor and clarity, the authors should consistently distinguish between (i) changes at the progenitor level (defined by appropriate markers) and (ii) patterns of final incorporation into differentiated tissues. Interpretations should be carefully tied to the level that is actually measured.
9. The authors examine muscle, peripheral neurons, and epidermis as examples of broadly distributed tissues. While the data suggest a lack of spatial precision in regenerative incorporation, the manuscript should be more cautious in interpreting these findings. For example, peripheral neurons exhibit broad incorporation, but this should be interpreted more conservatively as wound-adjacent amplification rather than a definitive test of tissue-identity dependence. Similarly, muscle and epidermis show some degree of spatially imprecise amplification, but this does not entirely exclude the possibility of feedback or surveillance mechanisms in response to tissue loss (Benham-Pyle et al., 2021, Kent et al., 2025).

Minor Comments:
1. Figure 1A: The authors should label injury types in Figure 1A for better readability.
2. Lines 104-105: It is unclear whether the authors used a limited number of z slices or the entire head for their analysis. How did they ensure proper alignment of different animals along the z axis?
3. Lines 185-186: How do the authors distinguish between high and low SMEDWI 1 expression? They may want to consider using a quantitative method to clarify this distinction.
4. Some images, such as Figures 2E, 2H, and S2C, are of poor quality and should be improved for better clarity.
5. Line 309 references "peripheral neurons (glipr 1$^+$, dd_210)"; however, Park et al. (2023) report that glipr 1 is a marker for parenchymal cells. The authors should reconcile this discrepancy or provide additional evidence supporting the assignment of glipr 1 expression to peripheral neurons in their dataset.
6. In Figure 6L, the epidermis shows almost no EdU positive cells at D+2. The authors should clarify whether this reflects a biological reality or a technical/analysis issue, and provide quantification or representative images to support their claim.
7. A summary table would greatly enhance the manuscript's readability, particularly by listing key details for each injury paradigm, such as tissue removal, proximity to analyzed tissues, regenerative outcomes, and whether GWR or MTR were induced.
8. The manuscript should include exact sample sizes in all figure legends.
9. The manuscript should clarify whether anesthesia treatments, such as chloretone or sodium azide, affect baseline proliferation or recovery in ways that might influence interpretation of the results.
10. The model in Figure 7 is relatively low in quality, and the labels are not sufficiently informative.

© 2026. Published by The Company of Biologists under the terms of the Creative Commons Attribution License (https://creativecommons.org/licenses/by/4.0/).

Reviewer 3

SUMMARY OF THE ADVANCE MADE IN THIS PAPER AND ITS POTENTIAL SIGNIFICANCE TO THE FIELD

In this manuscript, Pellegrini and Reddien investigate the specificity of progenitor responses to injury in the planarian Schmidtea mediterranea. The study addresses a longstanding question in regeneration biology, i.e. how organisms determine what tissues to regenerate following injury.

A common hypothesis in the field is that progenitor responses are adapted to the identity of the missing tissue. Instead, previous work from the same laboratory showed that eye regeneration in planarians does not rely on a specific increase in eye progenitor production, but rather on homeostatic progenitor production and reduced cell death (ref. 38). In the present manuscript, the authors extend this concept to several additional tissues, including the brain, multiple neuronal classes of the central nervous system, the ventral nerve cords, and the pharynx. The authors systematically compare injuries that remove specific tissues with injuries that occur nearby but leave those tissues intact. Based on these analyses, the authors propose that the identity of missing tissues does not strongly dictate the specificity of progenitor responses after injury. Instead, homeostatic cell turnover and injury-induced proliferation, informed by positional information, determines the identity of the cells produced at wound sites.
Overall, the manuscript presents a rigorous experimental testing of the specificity of tissue regenerative responses. However, several aspects of the analysis, terminology, and interpretation would benefit from clarification or additional discussion.

SUGGESTIONS TO AUTHORS

Major comments
- Fig. 1. Interpretation of EdU^{+} cells as a proxy for progenitor incorporation. The experiments presented in Fig. 1 rely on the assumption that the presence of EdU^{+} cells within the brain reflects incorporation of newly generated progenitors from outside the brain into the brain tissue. While this interpretation may be standard in the field of planaria regeneration, we find that the underlying assumptions are not clearly stated in the manuscript. It would be helpful to a broad readership if the authors explicitly described the reasoning linking EdU labeling, progenitor proliferation, differentiation and cellular incorporation.
- Related to the previous point, the manuscript would benefit from clearer definitions of terms used throughout the text, such as "incorporation," "proliferation," "amplification", "specification," and "differentiation".
- We find that the use of acronyms, for example GWR and MTR, makes the read cumbersome.
- Fig. S1C-D. We find the reference to Fig. S1C-D in line 117 difficult to interpret. These panels show H3P labeling, presumably to illustrate induction of the mitotic response, but these experiments were not clearly described in the text when quoted.
- Fig. 1F. I find the conclusion drawn from Fig. 1F and the associated paragraph (lines 121-127) somewhat difficult to follow. Is the observed brain bystander effect primarily driven by the incorporation of new neurons, glial cells, or both? Adding a short concluding statement summarizing the interpretation of this analysis would help readers understand the implications of the results.
- Fig. 2. The manuscript uses terminology such as dd_17258^{+}/dd_17258^{-}□ cells alongside with dd_17258 high / dd_17258 low cells (a similar issue arises for SMEDWI-1^{+}/ SMEDWI-1^{-}□ populations). It is not clear whether these terms refer to the same populations. Additionally, the criteria used to classify cells as "high" or "low" should be described in the Methods section.
- Fig. 2I-J. Lines 199-200: "This lack of response is consistent with the observed lower incorporation response of the inner cephalic ganglia arch described above (Fig. 1F right).". Fig. 2I-J shows no detectable differences in the number of gad^{+}/EdU^{+} or ppl-1^{+}/EdU^{+} neuronal cells for incision and AF/TF injury. Instead, Fig. 1F (inner arch region) shows a non-significant difference in the incorporation response for incision injury, but not for AF/TF injury. Could the author propose an explanation for the difference in response?
- Fig. 6K-L. The authors show images displaying differences in EdU^{+} cell distribution between dorsal and ventral epidermis. We invite the authors to provide quantification and statistical test for this difference.
- Throughout the manuscript, the authors use the term "trending" to describe cases in which multiple conditions are compared, differences are not significant, but they are in the same

© 2026. Published by The Company of Biologists under the terms of the Creative Commons Attribution License (https://creativecommons.org/licenses/by/4.0/).

direction. They seem to imply that these differences may be significant when examined together. I invite the author to apply a statistical test able to test significance for these joint comparisons.
- The authors propose that sustained proliferation after injury may amplify production of multiple progenitor types, including those corresponding to tissues that were not lost. We invite the authors to discuss what could happen to those cells that are amplified but do not match the missing tissue types.

Minor comments
-p-values labels could be defined once per legend or for the whole manuscript.
-Fig. 1A and Fig. 4A: please add labels identifying the different injury types (incision, TF injury, AF injury, head rim injury).
-Fig. 1A: a scale bar seems missing in the ChAT$^+$ neuron image.
-Supplementary Fig. 1 legend: "ANOVA" is misspelled.
-Fig. 1D legend: "EdU$^+$ cells / cephalic ganglion Z-slice" is unclear and it seems to not reflect what is plotted.
-Fig. 1E and Fig. 1F legends: please add descriptions of the figure panels.
-Fig. 1F schematic: clarify the meaning of the black dashed line in the legend. Why is it drawn only on one side of the schematic? Does it correspond to the "outer lobe slice" used in the quantification?
-Lines 118-119: the sentence "This injury also caused a significant increase in the incorporation of EdU into the brain" would be clearer as "This injury also caused a significant increase in the number of EdU$^+$ cells incorporated into the brain."
-Line 123: references appear to be incorrectly formatted.
-Line 142: "after anterior-flank animals" seems a typo.
-Fig. 1G and Fig. 1J: the incision schematic differs from the one shown in Fig. 1A and Fig. 1D. Please clarify whether this difference is intentional.
-Fig. 1I legend: "of" is missing.
-Fig. S2B legend: appears inconsistent with the panel. It may have been swapped with Fig. S2A. Additionally, the normalization per worm length should be described in the legend or in the Methods.
-Fig. S2B axis: if values represent cell number per worm length, the units should be mm^{-1}.
-Fig. 2I-J: here, the label is dpa instead of dpi. Is this intentional?
-Fig. S4A: arrows indicating the injury site are not visible despite being mentioned in the legend.
-Fig. 6M: consider adding titles for the different scenarios depicted in the schematics.
-Line 621: "were" is missing in "fixed worms were measured on coverslips".

First revision

Author response to reviewers' comments

Comments from the Reviewers:

Reviewer 1: SUMMARY OF THE ADVANCE MADE IN THIS PAPER AND ITS POTENTIAL SIGNIFICANCE TO THE FIELD

Pellegrini and Reddien addresses an important question in regeneration biology: how regenerative cell production is tailored to missing tissues. Using planarians and a set of carefully chosen "proximal but not ablative" injuries, the authors argue that injury identity has limited influence on progenitor output and that increased proliferation within broad fate-specification zones is sufficient to drive increased incorporation into multiple tissues, including uninjured ones ("bystander effect"). The work is conceptually clear and experimentally broad.

Thank you for the suggestions and thorough assessment of the work. We endeavored to address comments with text additions/modifications and new data as described below.

SUGGESTIONS TO AUTHORS

© 2026. Published by The Company of Biologists under the terms of the Creative Commons Attribution License (https://creativecommons.org/licenses/by/4.0/).

Major Comments
The two main points that, in my view, would most strengthen the paper are: (i) sharper separation of "incorporation" vs "net cell number / functional replacement" across tissues and time, (ii) additional controls/analyses that reduce alternative interpretations tied to axonal injury and systemic effects (apoptosis, remodeling).

1. "Incorporation" is not equivalent to "regenerative need" or "specific replacement"
Much of the evidence is based on EdU incorporation into marker-positive mature cells (and some progenitor markers), interpreted as "amplified production." This is valid for showing changes in lineage flux, but it does not always establish whether the tissue is being overproduced, maintained, or remodeled.

For each tissue class, explicitly state whether the claim is increased flux (EdU+ fraction/number) and/or increased mature cell number.
Where feasible, add or expand analyses of total cell numbers (or density) over time in the same structures in which incorporation is quantified (brain neuron subtypes, VNC cholinergic neurons, pharynx markers). The authors already provide limited total counts for dd_17258+ (supplement), but the interpretation remains cautious.

Thank you for your comments and suggestions. We have clarified the language throughout the text, being explicit when describing the incorporation of new cells into a mature cell population versus quantifying progenitors, and clarify that the claims are increased input.

We additionally added total counts for *th*+ cells in the brain and *npp-1*+ cells in the pharynx. We found no increase for *th*+ cells, and a modest increase for *npp-1*+ cells. These data were added to the paper (Figures S1C, S4D). Overall, when there is an increase in cell mature cell number it was not large despite increased new cell incorporation for populations assessed. This makes sense, because the missing tissue response includes increased cell death body wide. It is expected this would impact the mature cell numbers of all populations in this study, making any increase as a result of the bystander effect likely to be a transient one. Additionally, shifts in positional control gene expression during morphallaxis would change following most large injuries, eventually lowering production rate after the MTR. Regardless, future work could explore in more detail mature tissue abundance over time following different injuries, and the impact of cell death dynamics on this.

We added a section to the discussion to address some of the dynamics that are relevant to tissue abundance: "*The bystander effect could result in transient proportion changes between cells, but multiple important factors will impact relative cell numbers. The MTR triggers an increase in cell death that will push back on new cell addition outside of the wound. Furthermore, all tissues in planarians undergo turnover. It is unknown whether and how the incorporation rate of progenitors into a tissue impacts the death rate of that tissue. Regardless, once proliferation rates return to baseline after injury, turnover is expected to bring tissues, if out of proportion, back to steady state ratios (22).*"

2. Alternative interpretation: subtle injury to projections or local microdamage
The manuscript addresses this concern in multiple places (e.g., head tip injury for brain; esophagus incision for VNC), which is good. However, because the core claim challenges surveillance-like models, the standard for excluding "unnoticed damage" should be high.

For the brain bystander effect: consider adding an assay for axon integrity (e.g., pan-neuronal labeling of tracts) after each injury type used to argue "no brain damage," or include confocal views demonstrating that major tracts are intact.

The anterior flank injuries in the pre-pharyngeal do impact the VNCs when assessing for new cell incorporation into the brain. We therefore utilized a combination of approaches to further address this question beyond the head tip injury experiment used previously.

In addition to the head tip injury experiments, we assessed new cell incorporation into the brain following pharynx resection. Pharynx resection is a major injury that elicits a missing tissue response in the medial regions of the animal. The majority of axon tracts innervating

© 2026. Published by The Company of Biologists under the terms of the Creative Commons Attribution License (https://creativecommons.org/licenses/by/4.0/).

the pharynx originate in the VNCs as opposed to the brain, though there is some evidence of direct brain innervation. We find elevated incorporation of EdU-labeled cells into the brain in the case of pharynx resection as well.

Additionally, to further exclude the possibility that axon damage was creating a surveillance response, we compared a direct incision through the brain to a unilateral anterior flank injury, not impinging upon the brain. Incision did not cause an MTR- associated increase in neoblast proliferation whereas anterior-flank injury did. We found that direct brain incision is not sufficient to drive an increase in incorporation of new cells into the brain, but the unilateral anterior flank injury was - despite not injuring mature brain tissue. We would expect, if it was damage to axons that was driving the effect we observed, direct incision of the brain would have been sufficient to elicit a surveillance response. These conclusions are also supported by our pharynx results, where pharynx removal caused increased VNC production but direct bisection of the VNC (incision injury) did not. Finally, our data with 17258+ and *cintillo*+ head sensory neurons, which send axons to the brain but are not known to project into VNCs also support the conclusion that is not axon damage that was driving the bystander effect observed. These arguments and new pieces of data are now incorporated into the manuscript (see Figure 1 and associated text).

For pharynx removal and VNC incorporation: given that VNC bisection did not increase new ChAT+ cells but pharynx removal did, it would help to quantify proliferation spatial maps (or at least regional H3P) in the relevant segment for each condition to directly connect "proliferation near specification zones" to the effect size.

In the Figure 3F, we have provided an analysis of H3P+ cell counts to assess the mitotic activity in the area where VNC counting was performed, where an elevated number of mitoses was observed regional to the VNC for the pharynx flank condition but not VNC bisection.

Minor Comments
1. The epidermis section is one of the clearest arguments: blastema epidermis is derived largely from pre-existing post-mitotic progenitors; injury-induced epidermal amplification arrives later and broadly. This is convincing as presented, but it would help to connect these results more directly to the paper's main "specificity" thesis. However, make explicit whether epidermis represents an "exception that proves the rule" (i.e., turnover pipeline dominates acute repair) or a qualitatively different regulatory strategy.

We feel this connects to the specificity points in a nice way - the amplification of epidermis progenitors that occurs after injury arrives like troops after the battle is entirely over. This indicates that the amplification of progenitors that occurs is not geared towards replacing cells that are missing. This also helps demonstrate an advantage of the core turnover-based system of regeneration - in this case by providing progenitors produced before the system knew it would need them for an injury. We modified the discussion to make this point clearer. For example, this sentence was added: "*This points to a lack of specificity in the wound response, where the epidermal progenitors amplified by wounding are not made to replace the missing epidermis in regeneration*."

2. Define "bystander effect," "target-blind," "specificity," and "surveillance-like" early and use them consistently, ideally with a simple schematic that maps each to measurable outcomes. (Please note that bystander effect is also used in cell-ablation system).

We have added definitions where terms first appear, and removed "like" from "surveillance-like", as it more accurate is referred to simply as "surveillance" and we added a cartoon in Figure 1A to make the concepts clearer. Thank you for the suggestion.

AI Usage Declaration:

Reviewer's response in bullet points were uploaded to ChatGPT5.2 to improve grammar and text. No part of manuscript or abstract was uploaded.

© 2026. Published by The Company of Biologists under the terms of the Creative Commons Attribution License (https://creativecommons.org/licenses/by/4.0/).

Reviewer 2: SUMMARY OF THE ADVANCE MADE IN THIS PAPER AND ITS POTENTIAL SIGNIFICANCE TO THE FIELD

This manuscript by Pellegrini et al. addresses an important question in planarian regeneration: whether or not stem cell responses are specific to the identity of missing tissues after injury. Using EdU incorporation together with mature cell type markers, the authors examine multiple injury paradigms across several tissue systems and show that proximal injuries that spare mature tissues can nevertheless increase progenitor incorporation into spatially restricted neuronal populations. These data provide interesting insight into regenerative dynamics and suggest a bystander like mode of incorporation, and in many respects are conceptually consistent with the authors' earlier work (e.g. Wurtzel et al., 2015; Atabay et al., 2018).

SUGGESTIONS TO AUTHORS

However, I believe there are several conceptual issues that need to be addressed before the proposed model can be generalized and the conclusions fully supported. In particular, the choice and interpretation of markers for neural progenitors require more careful justification, and prior observations and models in the field that bear directly on this question are not sufficiently considered. Overall, I think the data should be interpreted more cautiously, and the manuscript would benefit from a clearer integration with existing literature.

Thank you for the thorough assessment as well as comments and suggestions. Points raised are addressed below, and we made a number of text edits and added new data to address several of the points.

Major points:
1. Line 160: The authors define "neural progenitors" as piwi1^{+}/pax6a^{+} cells. It is unclear whether this population corresponds to a clearly defined neural progenitor subset or broadly labels heterogeneous cell types. Pax6a is not a specific neural progenitor marker in planarians. The authors should include additional and more specific neural progenitor markers (e.g. soxB family members, hesl3) and, where possible, markers for defined neural progenitor subtypes (e.g. Wyss et al., 2023) to substantiate their claims.

We concur that *pax6a* expression marks multiple neural progenitor classes. To address this point we performed additional labeling experiments on a selection of neural markers (some broad and some more specific): *nkx2.1*, *otxA*, *sim*, and *tgs-1*. We find that all of the examined progenitors, as defined by expression of one of these markers, behaves similarly to *pax6a*+ cells: exhibiting an increase in number in relevant anatomical areas following proximal injury: all *transcription-factor+; smedwi-1*+ showed an increase by 5 dpi following anterior-flank, with *sim*+, *nkx2.1*+, and *otxA*+ cells also showing a significant increase at 3 dpi. This general behavior across multiple markers, we feel, provides substantial support for the observation made with *pax6a*. Of note, we feel that the strongest data is measurement of the actual new mature cell numbers present - determined with EdU for multiple specific populations (*cintillo*+, *dd_17258+*, *npp-1*+ pharyngeal neurons, and *phmus-1*+ pharyngeal muscle). We feel the specialized neoblast labeling provides support for this central method and shows an attendant increase in specialized neoblast progenitors. We moved the specialized neoblast data to figure 2, to have more of a supporting role and to give space for the new data added.

2. The authors propose an early, cross tissue "generic response" that is non specific with respect to tissue identity. If the early response is indeed an imprecise, progenitor level reaction, one would expect to detect a concomitant increase in other lineage progenitors in the same regions, not only in neurons. It is therefore unclear why comparable quantitative analyses were not performed for other lineages. Assessing proliferation of muscle, pharyngeal, intestinal, and epidermal progenitors using established lineage markers would be critical to determine whether these progenitor pools exhibit similar bystander like amplification, or whether the effect is in fact biased toward the neural lineage.

For this point, we added more progenitor data and the paper overall now includes muscle, pharynx, and epidermis progenitor data. There are several important considerations regarding this point:

© 2026. Published by The Company of Biologists under the terms of the Creative Commons Attribution License (https://creativecommons.org/licenses/by/4.0/).

1. We are proposing that there is indeed a generic response, where increased proliferation increases production of regional progenitors that are normally made in a region that is near the wound. Our focus for this study is on the bystander effect for regional tissues - those removed by some injuries but not by others. However, we are open to the possibility that not *all* specialized neoblasts will show the same generic behavior. For example, we considered in our work the possibility that uniform tissues will have evolved to show different behavior – e.g., our prior work had suggested that epidermal neoblasts were not as upregulated at wounds as were other neoblasts (van Wolfswinkel et al., 2014). However, we did in the end see evidence for upregulation of production for epidermis, muscle, and peripheral neurons (with interesting unique features) with injury. Nonetheless, whether all neoblasts are amplified to the same degree at wounds is an interesting problem, but not one we endeavored to answer in this work. It will remain an interesting problem for future work, especially as it relates to neoblast fate choice mechanisms. We improved our wording at the section of the paper where we transition to look at uniform tissues to help clarify the paper goals. For example: *" Beyond testing for the bystander effect with regional tissues and injuries that do not directly impact those regional tissues, we sought to explore other aspects of the response to injury and its specificity to the wound location by also studying more uniform tissues...... Do progenitors for broadly distributed tissues display different regenerative responses than do progenitors for regional tissues? Furthermore, if the production of broadly distributed cell types is amplified by injury is the amplification specifically targeted to the region injured or is there also increased production in the uninjured regions?"*

2. We are testing the bystander effect, where tissue production is upregulated when the tissue itself is not removed. This is not possible for muscle and epidermis. Nonetheless, we do test a related topic for these tissues – whether any upregulation of production that occurs is specific to the wound site or also occurs outside of the wound site. Note that we did not investigate the intestine in this work.

3. In this paper, we approached this question primarily by performing direct measurements of the number of new mature cell types present after injury: in other words, measuring EdU-labeled newly differentiated cell numbers. This is the most direct measurement of the number of functional new cell types produced. Measurements of specialized neoblast numbers are in of themselves important and informative, but we note that they represent an indirect measurement of differentiated cell production rate. For instance, there could be more specialized neoblasts made transiently that do not go on to differentiate or otherwise fail to integrate into their target tissues. But when more EdU- labeled differentiated cells are present, there is little ambiguity that the number of new, injury induced mature cells went up. As such, we measured muscle, pharynx, and epidermis production directly with EdU experiments as our primary approach. Nonetheless we also performed additional experiments with neoblast progenitors. The upregulation of pharynx progenitors by certain injuries at least has been previously well documented (Bohr et al., 2021). We did additional experiments with this, but the *FoxA* probe repeatedly resulted in very poor staining in our hands, and gives rise to a varied complement of pharyngeal cells with potentially different differentiation or turnover dynamics. dd_554, which marks a pharynx progenitor population, proved to be a very specific and much more robust probe, and our data with this probe strongly supports a conclusion that our bystander observations with the pharynx involve increased progenitor production (Figure 4G-H). We also measured muscle specialized neoblasts *(myoD*, *snail)+/smedwi-1+* – we did not see increased numbers between brain lobes but did see more of these cells at the wound. Therefore, more EdU labeling muscle in the wound is consistent with more specialized muscle neoblasts present there. We also note an overall increase in (EdU-labeling measured) new mature muscle cells at and just proximal to the wound, with a lower amount distal to the wound. This raises interesting questions for future work related to muscle specialized neoblast production pattern and possible migratory recruitment of post-mitotic progenitors, and we note this in the text.

Prior work has assessed the behavior of *zfp-1+* (epidermal) neoblasts in response to wounding, and demonstrated a lower response compared to broader neoblast classes (van Wolfswinkel, 2014). Instead, they show an increase in early post-mitotic *prog-2+* epidermal progenitors at the wound. Considering this reviewer comment, we decided to add more data to this version

© 2026. Published by The Company of Biologists under the terms of the Creative Commons Attribution License (https://creativecommons.org/licenses/by/4.0/).

of the manuscript on epidermal progenitor numbers in the context of the injuries used. We used *agat-3*+ late epidermal post-mitotic progenitors, and we found that an increase in the numbers of these cells 96hpi following anterior-flank injury and a spatial bias of these cells towards the wound; consistent with prior data and the EdU-based observations on the epidermis presented here.

3. The manuscript overlooks the heterogeneity of neoblast and progenitor populations, particularly tgs1+ neoblasts and other progenitors, which have been shown to both respond to amputation (Zeng et al., 2018) and may contain neural progenitors (Raz et al., 2021). The authors should clarify how tgs1+ neoblasts behave during regeneration and whether the progenitor response is a general neoblast injury induced reaction or reflects a more specialized response from the regenerating sensory nervous system. The possibility that injury responsive tgs1+ neoblasts or sensory neurons may serve as part of a surveillance mechanism should be explicitly considered and addressed in the manuscript.

We added an analysis of *tgs-1*+ neoblast behavior to Figure 2. As the reviewer notes, our prior work indicates that *tgs-1*+ neoblasts are neural specialized and we concur that some wound-induced gene expression can occur in these neural neoblasts. We see that *tgs-1*+ neoblasts are amplified by wounding by 5dpi with similar magnitude as the other neural specialized neoblasts examined (Figure 2 K, L). We don't at present have more to add regarding the behavior of *tgs-1*+ neoblasts beyond noting that they behave similarly to other markers used.

4. In addition, post mitotic injury induced cell states that arise in response to damage was observed in Benham Pyle et al. (2021). Tgs1 staining showed a significant increase in pluripotent neoblasts after injury (Zeng et al., 2018). The pax6a^{+} cells quantified here could, at least in part, correspond to such injury induced states rather than bona fide neural progenitors. The manuscript should consider and explicitly discuss this possibility, and clarify how the proposed model of non specific progenitor responses is reconciled with existing models involving post mitotic injury induced cell states and pluripotent neoblast expansion.

First, we now cite Benham Pyle (2021) in the discussion and note that injury induced states exist in different tissues and that these are of interest for regenerative responses in general. The reviewer suggests that increased *pax6a*+ cells could potentially represent a transient injury-induced state rather than neural progenitors. In injury response timecourse data, pax6a shows no sign of being a wound-induced gene (Wurtzel 2015; see below image comparing expression for all TFs used in a time course, compared to a wound-induced gene *wnt1* in hours after injury; *pax6a* levels, and the levels for other TFs used, only increase when new neural specialized neoblasts are appearing during neoblast division amplification in the MTR ~48hpa; https://radiant.wi.mit.edu/app/). We added this new information to the supplement (Figure S2E).

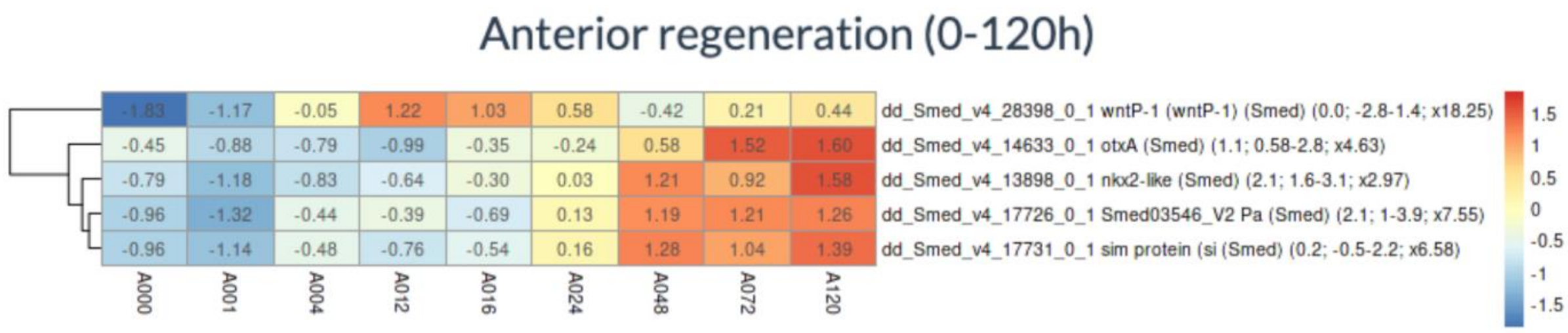

To further address the reviewer's concern regarding whether this is merely a generic, non-specific transcriptional state, we assessed a panel of additional neural progenitor markers: *tgs-1*, *otxA*, *sim*, and *nkx2.1*. All show an increase in response to the anterior-flank injury, suggesting that even if the *pax6a*+ spike were simply reflective of isolated, transient transcriptional noise or a generic injury state, it is highly unlikely that such a program would coordinately and selectively upregulate a whole suite of distinct neural lineage markers.

Regardless, we also note that our primary measurement approach is an increase in EdU-labeled new differentiated cells in mature tissue (e.g., brain), which come from neoblasts and

© 2026. Published by The Company of Biologists under the terms of the Creative Commons Attribution License (https://creativecommons.org/licenses/by/4.0/).

these EdU numbers cannot be explained through a transient wound-induced state that does not impact production of progenitors. Transient injury states occur independently of stem cell division and resolve without appearing to generate new differentiated cell lineages (Benham-Pyle et al., 2021). Our study primarily assesses the functional incorporation of these cells into mature neural tissue. Taken together, these data indicate a production of bona fide neural progenitors resulting in new cell incorporation, rather than a transient cell state. With regard to pluripotent neoblast expansion, this project does not investigate or address models of neoblast pluripotency/lineage; we are hesitant to frame wording on pluripotent neoblast states/expansion in this paper. We note that our prior work shows that non-*tgs-1*+ neoblasts can make colonies (Raz 2021 Fig. 6), that more than half of all 4-cell colonies do not have a single *tgs-1*+ cell (Raz 2021, Fig. 5B), that there is no other specialized neoblast cluster that contains neural neoblasts than the cluster with *tgs-1*+ cells (King 2024), and that the *tgs-1*+ neoblast cluster is enriched entirely for neural FSTFs and neural differentiated markers (Raz 2021). Based on this evidence in our other work, and the fact that this paper does not investigate this topic further (we did add *tgs-1*+ cell counts) we chose to not go in depth in the text on the topic of neoblast pluripotency.

5. Several key claims rely on bulk quantification of EdU$^+$ cells as evidence for generic amplification. This metric alone cannot distinguish lineage specific from lineage agnostic responses. A more informative approach would be to quantify multiple lineage specific progenitor markers in parallel within key "bystander" regions (e.g. dd_554$^+$ zones) at early post injury time points. Demonstrating coordinated amplification across unrelated progenitor pools, rather than in a single lineage, would provide much stronger support for a generic response model. Moreover, increased incorporation of EdU$^+$ cells does not necessarily indicate that progenitor specification is non specific. The final count of EdU$^+$ mature cells may reflect various processes, including proliferation, fate specification, local retention, survival, and differential integration. The authors should at least discuss these caveats.

The reviewer uses a particular framing here – lineage-specific and lineage agnostic responses that is somewhat in line with our work but not in perfect alignment with our intent (see reply to point 2 above). We are simply assessing whether injury near regional tissues that are not lost to injury upregulates the production of those tissues (the bystander effect). This is primarily tested for regional tissues; uniform tissues will be removed with all injury types involving missing tissue. We do not know whether all fate specification frequencies will scale equally following injury-induced proliferation, though in principle they could. Nonetheless, we do add more progenitor quantification FISH experiments to this version of the manuscript as noted above, that complement in particular our quantifications of a pharyngeal dd_554 progenitor response following an independent injury protocol that completely leaves the pharynx intact.

In both distinct experimental contexts, the uninjured "bystander" progenitor pool significantly amplified and proceeded to incorporate into its respective mature tissue. We argue that if these regenerative responses were strictly lineage-specific or guided by localized tissue-loss feedback loops, an injury that spares the pharynx should not provoke a pharyngeal progenitor response, nor should a brain-sparing injury provoke a neural one. Seeing this consistent behavior across completely different organ-specific contexts strongly suggests a generalized, systemic wound response that operates independently of local tissue damage.

Regarding the reviewer's point on processes that could impact EdU number, we concur that a higher final count of EdU$^+$ mature cells could in principle be impacted by (i) a change in number of fate-specified neoblasts made but also (ii) by more survival/integration of progenitors made and this is therefore a reasonable point for consideration of wording and interpretation: we deem that both changes in cell proliferation or fate specification would affect progenitor number (Of note, even if progenitor production was not responsible for increased incorporation of EdU+ cells into mature tissue, this would still represent a bystander effect where the increased numbers occur for non-injured tissues.) To focus on survival/integration further however: There is no evidence of substantial cell death happening normally in progenitor pools. For example, lethal irradiation does not overtly change the homeostatic number of cell death events (TUNEL-labeling – see Pellettieri et al.,

© 2026. Published by The Company of Biologists under the terms of the Creative Commons Attribution License (https://creativecommons.org/licenses/by/4.0/).

2010). If there was an excess fraction of neoblast progeny that did not normally incorporate into mature tissue that was culled, as would be the case if our data were the result of increased survival, then then this would be expected to contribute substantially to number of homeostatic cell deaths in the animal given the immense cell division that continually occurs. We also show that the bystander effect correlates spatially and with injury-type in cases where increased neoblast proliferation occurs. We suggest that the most parsimonious explanation for increased EdU+ cell number in a differentiated tissue is more production of progenitors given these considerations combined with the observations of more eye progenitors produced after anterior-flank injuries in LoCascio 2017, more dd_554+ progenitors in this work, and more *pax6a*, *nkx2.1*, *otxA*, *sim*, and *tgs-1* positive neoblasts after flank injuries also in this work.

Nonetheless, we selected wording on EdU results carefully throughout the manuscript to refer to increased new cell incorporation to a differentiated tissue and added note on this caveat and the relevant considerations as discussed above to the manuscript.

" Much of our work utilized the number of new mature cells (EdU+ cells derived from neoblasts). This number could in principle be affected not just by progenitor-production increase, but also by other changes including increased progenitor survival above baseline. However, neoblasts and their progeny do not overtly contribute to homeostatic cell-death levels (20); as would be expected if injury was causing a reduction in homeostatic progenitor death. Furthermore, neoblast proliferation increases correlated with observed bystander effects throughout this work, eye progenitors are amplified by injuries outside of the eye (38), and we observed increased numbers of many neural neoblast classes and pharynx progenitors in bystander experiments. These considerations support a model where increased regional neoblast proliferation increases new tissue production (Figure 7B)."

6. Line 116: The head tip injury may also affect mechanosensory neurons (Ross et al., 2018), potentially influencing the VNCs. Therefore, the data do not fully rule out the possibility that the observed bystander effect in the brain could result from surveillance based detection of damage by sensory neurons. This possibility should be explicitly discussed, and the authors should clarify to what extent their data distinguish between a generic cross tissue response and a sensory neuron-mediated surveillance mechanism.

We decided to add additional experiments to address this comment, comparing a direct incision to the brain to a unilateral anterior flank. We note that even in a sensory-neuron injury detection system new cell production would be occurring for non-removed tissues (e.g., pharynx muscle following flank cut, etc.). Should surveillance-based detection of damage by sensory neurons drive the observed bystander effect, we'd expect such a brain incision to cause a comparable or greater increase in incorporation as compared to a unilateral anterior flank injury. Instead, we observe no significant increase in incorporation following direct brain incision, suggesting there was not a detectable sensory neuron-mediated surveillance mechanism informing our previous data. While both injuries do impact neurons, the brain incision impacts both the cephalic ganglia and the lateral sensory neuron apparatus directly. That we do not observe a response to brain incisions, which do not elicit increased neoblast proliferation, suggests that our data is more indicative of a generic regenerative response than surveillance mediated by sensory neuron damage.

We have amended Figure 1 and the manuscript to incorporate these new data.

7. The manuscript's central conclusion seems overstated relative to the data presented and the broader context of the field. While the study demonstrates broad injury-associated amplification and bystander-like incorporation, the title and several summary statements (e.g., lines 538-542) overstate the claim that the identity of the missing tissue has minimal impact on determining stem cell response specificity. This assertion should be moderated. Targeted regeneration studies support a more nuanced perspective that acknowledges both "specific" and "non-specific" regeneration (Bohr et al., 2021; Vu et al., 2015). A more defensible framing would emphasize that generic amplification and bystander incorporation are important components of regeneration, without dismissing identity based specificity. The title, abstract, and discussion should be revised accordingly and explicitly limited to planarians.

© 2026. Published by The Company of Biologists under the terms of the Creative Commons Attribution License (https://creativecommons.org/licenses/by/4.0/).

We concur that our work does not exclude the possibility of both targeted (specific) and non-specific responses, which we did endeavor to note in the original version. Given this reviewer point, however, we reassessed our wording and modified language in attempt to make this compatibility more explicit. For example, in the discussion: *" In principle, some tissues might layer onto this generic mechanism a surveillance system that enhances the specificity and/or magnitude of the response provided. For instance, evolution could select for a mechanism that senses damage for tissues that are exposed to the environment and injured frequently (e.g., potentially the pharynx). Such surveillance added to the generic mechanism is fully compatible with bystander effects still occurring." At the end of the introduction: " Overall, our findings indicate that the identity of missing tissues does not strongly dictate regenerative outcome, although the general process we describe could in principle be coupled with a surveillance process added for certain tissues."*

We have also revised the title, abstract, and discussion to make clear this study focuses on planarians.

8. It appears that the authors interpret the results of "increased EdU incorporation into mature cell types" as broader statements regarding "nonspecific stem cell/progenitor responses." If this is the case, why not directly quantify muscle progenitors using established markers (e.g., MyoD) in figures such as Figures 5A and G-J? The same reasoning applies to Figures 6C-J, where co-staining and direct counts of epidermal progenitor populations would offer further clarity. For enhanced rigor and clarity, the authors should consistently distinguish between (i) changes at the progenitor level (defined by appropriate markers) and (ii) patterns of final incorporation into differentiated tissues. Interpretations should be carefully tied to the level that is actually measured.

Our initial approach targeted mature tissue incorporation as the most functional and direct readout of regenerative outcomes - integration into the mature tissue. However, we have amended language in the manuscript to more carefully delineate between changes at the progenitor level and pattern of final incorporation. Furthermore, we directly quantified *(myoD, snail)+/smedwi-1+* muscle progenitors and *agat-3+* post-mitotic epidermal progenitors and have amended the data into the relevant Figures 5 and 6 supplement respectively.

9. The authors examine muscle, peripheral neurons, and epidermis as examples of broadly distributed tissues. While the data suggest a lack of spatial precision in regenerative incorporation, the manuscript should be more cautious in interpreting these findings. For example, peripheral neurons exhibit broad incorporation, but this should be interpreted more conservatively as wound-adjacent amplification rather than a definitive test of tissue-identity dependence. Similarly, muscle and epidermis show some degree of spatially imprecise amplification, but this does not entirely exclude the possibility of feedback or surveillance mechanisms in response to tissue loss (Benham-Pyle et al., 2021, Kent et al., 2025).

We agree with this point, and we did not intend in our original submission to imply that these tissues are tests of the bystander effect - indeed we were interested to study how production of these tissues fared because they are distinct from regional tissues, being lost with all large injury types. We have been curious about the behavior of such broadly distributed tissues for many years, following initial observations in van Wolfswinkel 2014 that epidermal neoblasts amplified less at wounds than other neoblasts, and given the considerations regarding epidermal differentiation time implying that increased epidermal neoblast production could not realistically contribute to blastema formation. We can readily see how the transition to this part of the manuscript could be unclear, or could be perceived to be testing for absence of surveillance. We therefore modified the transition to this section to make it clearer: *" Beyond testing for the bystander effect with injuries that do not directly impact regional tissues, we explored the specificity of cell-type production to wound location by studying more uniform tissues...... Does new cell production for broadly distributed tissues display different regenerative responses than for regional tissues? Furthermore, if the production of broadly distributed cell types is amplified by injury are new cells specifically targeted to the injured region, or also to uninjured regions?"*

© 2026. Published by The Company of Biologists under the terms of the Creative Commons Attribution License (https://creativecommons.org/licenses/by/4.0/).

Regarding the observations, we found it of interest that we observed increased incorporation into non-injured regions, consistent with a general lack of specificity in the regenerative outcome for these tissues. However, we cannot determine whether the increased production was initially associated with detecting damage to these tissues or not.

Minor Comments:
1. Figure 1A: The authors should label injury types in Figure 1A for better readability.
Labels have been added.
2. Lines 104-105: It is unclear whether the authors used a limited number of z slices or the entire head for their analysis. How did they ensure proper alignment of different animals along the z axis?
We added language to the Methods and Material section to fully describe selection of the singular Z-slice utilized for analysis, and clarified in the manuscript and figure legends we quantified in a single z-slice.
3. Lines 185-186: How do the authors distinguish between high and low SMEDWI 1 expression? They may want to consider using a quantitative method to clarify this distinction.
The distinction was qualitative during data analysis, and was originally pointed out to reflect the process of differentiation (SMEDWI-1 protein perdurance decreases with time as differentiated marker expression increases). It is not, however, a quantitatively useful distinction and we have removed it.
4. Some images, such as Figures 2E, 2H, and S2C, are of poor quality and should be improved for better clarity.
We have replaced Figures 2H and S2C with higher quality representatives of *th*+ neurons. Figure 2E reflects the general quality of the SMEDWI-1 protein stain as analyzed and counted, and we have not "cleaned up" the background signal in any image presented.
5. Line 309 references "peripheral neurons (glipr 1^{+}, dd_210)"; however, Park et al. (2023) report that glipr 1 is a marker for parenchymal cells. The authors should reconcile this discrepancy or provide additional evidence supporting the assignment of glipr 1 expression to peripheral neurons in their dataset.
Park et al., (2023) refers to dd_924 as "glipr-1+", whereas we refer to dd_210 as "glipr-1+". Both dd_IDs BLAST to GLI pathogenesis related- 1, presumably informing their naming convention and informing our own. We have added a citation showing the glipr-1+, dd_210 cells we quantify are characterized as peripheral neurons and named "*glipr-1*" in Fincher at al., (2018).
6. In Figure 6L, the epidermis shows almost no EdU positive cells at D+2. The authors should clarify whether this reflects a biological reality or a technical/analysis issue, and provide quantification or representative images to support their claim.
In Figure 6L, animals were fixed 5dpi following EdU delivery at D+2. In Figure 6B, animals were fixed 6dpi following EdU delivery at D+2. In Figure 6B (right), we show there is an abundance of EdU in the blastema overall, but not in that blastema's epidermis, demonstrating the lack of epidermal EdU is not due to technical or analytical issue.

We added quantifications for the images in Figure 6L in Figure 6M.
7. A summary table would greatly enhance the manuscript's readability, particularly by listing key details for each injury paradigm, such as tissue removal, proximity to analyzed tissues, regenerative outcomes, and whether GWR or MTR were induced.

We have provided a summary table in Table S2.
8. The manuscript should include exact sample sizes in all figure legends.

We have provided minimum n numbers for each figure panel in the respective figure legends, and exact n-numbers for each condition in each experiment in Table S1.
9. The manuscript should clarify whether anesthesia treatments, such as chloretone or sodium azide, affect baseline proliferation or recovery in ways that might influence interpretation of the results.
We have clarified in the Methods and Material section the effects of both treatments on baseline proliferation or recovery in so far as the information is available. Additionally, comparison of untreated controls to treated uninjured animals showed no measurable differences in the metric assessed (EdU+/*ChAT*+ cells in VNC), in Figure S3C.
10. The model in Figure 7 is relatively low in quality, and the labels are not sufficiently informative.

© 2026. Published by The Company of Biologists under the terms of the Creative Commons Attribution License (https://creativecommons.org/licenses/by/4.0/).

To address this suggestion, we submitted a description of the model as well as the received feedback to a graphic designer for input and improvement, and have amended Figure 7B to include this newer version with modifications from this graphical designer.

Reviewer 3: SUMMARY OF THE ADVANCE MADE IN THIS PAPER AND ITS POTENTIAL SIGNIFICANCE TO THE FIELD

In this manuscript, Pellegrini and Reddien investigate the specificity of progenitor responses to injury in the planarian Schmidtea mediterranea. The study addresses a longstanding question in regeneration biology, i.e. how organisms determine what tissues to regenerate following injury.

A common hypothesis in the field is that progenitor responses are adapted to the identity of the missing tissue. Instead, previous work from the same laboratory showed that eye regeneration in planarians does not rely on a specific increase in eye progenitor production, but rather on homeostatic progenitor production and reduced cell death (ref. 38). In the present manuscript, the authors extend this concept to several additional tissues, including the brain, multiple neuronal classes of the central nervous system, the ventral nerve cords, and the pharynx. The authors systematically compare injuries that remove specific tissues with injuries that occur nearby but leave those tissues intact. Based on these analyses, the authors propose that the identity of missing tissues does not strongly dictate the specificity of progenitor responses after injury.
Instead, homeostatic cell turnover and injury-induced proliferation, informed by positional information, determines the identity of the cells produced at wound sites.
Overall, the manuscript presents a rigorous experimental testing of the specificity of tissue regenerative responses. However, several aspects of the analysis, terminology, and interpretation would benefit from clarification or additional discussion.

Thank you for the thorough assessment of the paper, as well as the support and suggestions, which we endeavored to address below.

SUGGESTIONS TO AUTHORS
Major comments
- Fig. 1. Interpretation of EdU$^+$ cells as a proxy for progenitor incorporation. The experiments presented in Fig. 1 rely on the assumption that the presence of EdU$^+$ cells within the brain reflects incorporation of newly generated progenitors from outside the brain into the brain tissue. While this interpretation may be standard in the field of planaria regeneration, we find that the underlying assumptions are not clearly stated in the manuscript. It would be helpful to a broad readership if the authors explicitly described the reasoning linking EdU labeling, progenitor proliferation, differentiation and cellular incorporation.

Thank you for this suggestion. We can see how this key framing information could be more clearly presented. We have added language to more clearly describe the use of EdU+ cell incorporation and the biology underlying the process. We explicitly describe that stem cell division is not known to occur within organs in planarians (there is no evidence of organ-specific neoblasts residing within organ structures), such that all new cells derive from mesenchymal neoblasts, which are the only somatic dividing cells. They produce progeny cells that migrate and incorporate into tissues where they differentiate. We also added a cartoon to Figure 1A to graphically illustrate this key biological framing for the work.
- We find that the use of acronyms, for example GWR and MTR, makes the read cumbersome.

We considered your comment carefully, and decided to eliminate the use of GWR to make things simpler in the text, and to rely only on MTR for brevity. We also eliminated FSTF (outside of the definition for connection to other literature), AF (anterior-flank), and TF (tail-flank).

- Fig. S1C-D. We find the reference to Fig. S1C-D in line 117 difficult to interpret. These panels show H3P labeling, presumably to illustrate induction of the mitotic response, but these experiments were not clearly described in the text when quoted.

We include those data to demonstrate that the head tip injury induces a mitotic response 48 hpi, as has previously been shown (Wenemoser and Reddien, 2010), and we modified the text.

© 2026. Published by The Company of Biologists under the terms of the Creative Commons Attribution License (https://creativecommons.org/licenses/by/4.0/).

- Fig. 1F. I find the conclusion drawn from Fig. 1F and the associated paragraph (lines 121-127) somewhat difficult to follow. Is the observed brain bystander effect primarily driven by the incorporation of new neurons, glial cells, or both? Adding a short concluding statement summarizing the interpretation of this analysis would help readers understand the implications of the results.

We cannot preclude that incorporation of new glial cells might be contributing to the bystander effect, however our analysis of the nuclei-rich regions of the cephalic ganglia demonstrates that new neural incorporation is a major component of the brain bystander effect, and thus can be considered reflective of new neuron incorporation dynamics. We added a short concluding statement to help clarify what is now Figure 1I: "*We conclude then that the previously observed brain bystander effect involves new neuron production.***"**

- Fig. 2. The manuscript uses terminology such as dd_17258$^+$/dd_17258$^-$ cells alongside with dd_17258 high / dd_17258 low cells (a similar issue arises for SMEDWI-1$^+$/ SMEDWI-1$^-$ populations). It is not clear whether these terms refer to the same populations. Additionally, the criteria used to classify cells as "high" or "low" should be described in the Methods section.

We decided to remove the "high" or "low" classifications, as both dd_17258 high/SMEDWI-1 low and dd_17258 low/SMEDWI-1 high refer to the same cell population but in qualitatively distinct stages of differentiation: SMEDWI-1 protein levels decline over time as the cell differentiates (*smedwi-1* transcription ceases when cells leave the neoblast state and SMEDWI-1 protein gradually degrades). This delineation was found to not really be necessary for the point made, as there was no material difference between these populations that we can determine, so we removed this to avoid confusion.

- Fig. 2I-J. Lines 199-200: "This lack of response is consistent with the observed lower incorporation response of the inner cephalic ganglia arch described above (Fig. 1F right).". Fig. 2I-J shows no detectable differences in the number of gad$^+$/EdU$^+$ or ppl-1$^+$/EdU$^+$ neuronal cells for incision and AF/TF injury. Instead, Fig. 1F (inner arch region) shows a non-significant difference in the incorporation response for incision injury, but not for AF/TF injury. Could the author propose an explanation for the difference in response?

The difference in response is likely explained by power – both *ppl-1+ and gad+* populations contain a relatively small number of neurons each, especially compared to the number of nuclei in the cephalic ganglia arches. We posit that the decreased incorporation of the inner arch region compared to the outer arch region may point to differences in the turnover dynamics of neurons in the medial region (like *ppl-1+* and *gad+* neurons). However, whether this holds true and it is the case that our study was underpowered to observe the effect for *ppl-1+* and *gad+* neurons, or whether there is a stratification of responses within neural progenitor classes remains unclear – we have added language to make this more explicitly clear.

- Fig. 6K-L. The authors show images displaying differences in EdU$^+$ cell distribution between dorsal and ventral epidermis. We invite the authors to provide quantification and statistical test for this difference.

We have provided manually counted quantifications with accompanying statistical tests in Figure 6M, scored blind to condition.

- Throughout the manuscript, the authors use the term "trending" to describe cases in which multiple conditions are compared, differences are not significant, but they are in the same direction. They seem to imply that these differences may be significant when examined together. I invite the author to apply a statistical test able to test significance for these joint comparisons.

We agree with the reviewer's comment on wording and removed the word "trending" and replaced this with "not significant". Our original study intent was to evaluate each experimental condition relative to its control at that timepoint independently, as such, we decided to retain the use of t-tests. We did additionally performed pooled analyses however,

© 2026. Published by The Company of Biologists under the terms of the Creative Commons Attribution License (https://creativecommons.org/licenses/by/4.0/).

which overall concur with our current interpretations and statistical models: pooling across time points yields a significant overall injury effect for several of these comparisons (e.g., Fig. 5F), while for others the injury effect is weaker or time-dependent.

Panel	Population (region)	Model	Injured/control ratio	95% CI	p (pooled)	Condition × time-point p
C	glipr-1⁺, whole area	GLM (Tweedie)	1.78	1.35–2.33	<0.001	0.341
D	glipr-1⁺, wound-distal	GLM (Tweedie)	1.47	0.99–2.18	0.059	0.154
E	glipr-1⁺, wound-proximal	GLM (Tweedie)	1.39	0.93–2.05	0.105	0.026
F	glipr-1⁺, in-blastema	GLM (NB)	2.94	1.13–7.62	0.027	0.811
G	colF-2⁺, whole area	GLM (Tweedie)	1.07	0.91–1.27	0.410	0.047
H	colF-2⁺, wound-distal	GLM (Tweedie)	0.83	0.63–1.08	0.171	0.047
I	colF-2⁺, wound-proximal	GLM (Tweedie)	1.30	0.94–1.79	0.112	0.084
J	colF-2⁺, in-blastema	GLM (NB)	4.67	2.87–7.60	<0.001	0.017

Injured/control ratios (>1: more incorporation in injured animals) are the overall effect of injury from a generalized linear model with injury status and time point as factors (cell-means parameterization), pooled (averaged) across the three time points (D8, D11, D16).

Condition × time-point p tests whether the injury effect varied across time points. Where significant (E, G, H) the pooled ratio summarizes an effect that changes over time (e.g., colF-2⁺ wound-distal decreases significantly by D16: ratio 0.54, p = 0.015; see the per-time-point sheet).

- The authors propose that sustained proliferation after injury may amplify production of multiple progenitor types, including those corresponding to tissues that were not lost. We invite the authors to discuss what could happen to those cells that are amplified but do not match the missing tissue types.

Thank you for this question - we felt it would add to the paper to address this in the discussion. Our findings indicate that increased progenitor production leads to increased presence of newly differentiated cells. Concurrently, there will be increased cell death body wide associated with the missing tissue response. Importantly, there is continual turnover of all differentiated cells of the body. Over time, when proliferation is back to the basal level, turnover will bring cell populations back to steady state ratios (see Reddien 2024 for discussion) - even for any tissues that have become out of proportion with other tissues because of injury responses. We added some discussion of this topic to the discussion section of the paper: "*The bystander effect could result in transient proportion changes between cells, but multiple important factors will impact relative cell numbers. The MTR triggers an increase in cell death that will push back on new cell addition outside of the wound. Furthermore, all tissues in planarians undergo turnover. It is unknown whether and how the incorporation rate of progenitors into a tissue impacts the death rate of that tissue. Regardless, once proliferation rates return to baseline after injury, turnover is expected to bring tissues, if out of proportion, back to steady state ratios (22).*"

Minor comments
-p-values labels could be defined once per legend or for the whole manuscript.
We have defined p-values once per legends
-Fig. 1A and Fig. 4A: please add labels identifying the different injury types (incision, TF injury, AF injury, head rim injury).
We have added descriptive labels to the injuries
-Fig. 1A: a scale bar seems missing in the ChAT⁺ neuron image.
Image was removed to add diagrams to clarify neoblast biology
-Supplementary Fig. 1 legend: "ANOVA" is misspelled.
Thank you, it was corrected
-Fig. 1D legend: "EdU⁺ cells / cephalic ganglion Z-slice" is unclear and it seems to not reflect what is plotted.
We clarified that the cephalic ganglion z-slice represents the counting area (as diagramed in the respective figure).
-Fig. 1E and Fig. 1F legends: please add descriptions of the figure panels.

We added more description
-Fig. 1F schematic: clarify the meaning of the black dashed line in the legend. Why is it drawn only on one side of the schematic? Does it correspond to the "outer lobe slice" used in the

© 2026. Published by The Company of Biologists under the terms of the Creative Commons Attribution License (https://creativecommons.org/licenses/by/4.0/).

quantification?
We added language to the legend to clarify the black dashed line is the counting area within a single z-slice of the image. It is drawn only on one side because quantifications were performed only on 1 of the two cephalic ganglions.
-Lines 118-119: the sentence "This injury also caused a significant increase in the incorporation of EdU into the brain" would be clearer as "This injury also caused a significant increase in the number of EdU^+ cells incorporated into the brain."
Thank you, we have clarified such language throughout the manuscript
-Line 123: references appear to be incorrectly formatted.
We have corrected this, thank you for pointing it out
-Line 142: "after anterior-flank animals" seems a typo.
We have corrected this, thank you for pointing it out
-Fig. 1G and Fig. 1J: the incision schematic differs from the one shown in Fig. 1A and Fig. 1D. Please clarify whether this difference is intentional.
We modified the incision schematic to be the same. The incision injuries were all carried out identically, the difference was unintentional.
-Fig. 1I legend: "of" is missing.
Amended, thank you.
-Fig. S2B legend: appears inconsistent with the panel. It may have been swapped with Fig. S2A. Additionally, the normalization per worm length should be described in the legend or in the Methods.
It was swapped, we corrected it. We added language to the Materials and Methods section to describe the normalization per worm length.
-Fig. S2B axis: if values represent cell number per worm length, the units should be mm^{-1}.
Thank you, corrected.
-Fig. 2I-J: here, the label is dpa instead of dpi. Is this intentional?
No, it has been amended.
-Fig. S4A: arrows indicating the injury site are not visible despite being mentioned in the legend.
Amended, thank you.
-Fig. 6M: consider adding titles for the different scenarios depicted in the schematics.
We added titles for the different scenarios, now Figure 7A.
-Line 621: "were" is missing in "fixed worms were measured on coverslips".
Amended, thank you.

Second decision letter

MS ID#: dev.205573R1

MS TITLE: Lack of specificity of planarian progenitor responses to injury in regeneration

AUTHORS: Cecilia Pellegrini; Peter W Reddien

Dear Peter,

I have now received all the referees reports on the above manuscript, and have reached a decision. The referees' comments are appended below.

The overall evaluation is positive and we would like to publish a revised manuscript in Development, provided that Reviewer 2 comments can be satisfactorily addressed. Please attend to these comments in your revised manuscript and detail them in your point-by-point response. The comments are minor and we do not expect to send the revised version back to peer review.

Reviewer 1

The authors have satisfactorily addressed all the issues raised by the reviewer.

© 2026. Published by The Company of Biologists under the terms of the Creative Commons Attribution License (https://creativecommons.org/licenses/by/4.0/).

Reviewer 2

SUMMARY OF THE ADVANCE MADE IN THIS PAPER AND ITS POTENTIAL SIGNIFICANCE TO THE FIELD

The revised manuscript is strengthened by the new analyses of neural-associated neoblasts and progenitors of other lineages. Several issues of interpretation remain.

SUGGESTIONS TO AUTHORS:

1. The interpretation of tgs-1$^+$ neoblasts as neural progenitors remains overstated. The new data show that tgs-1$^+$/smedwi-1$^+$ cells increase after anterior-flank injury. This establishes amplification of a tgs-1-expressing neoblast population, but not its developmental output. The manuscript describes tgs-1$^+$ cells as "neural-fate-enriched neoblasts." This wording should be retained. By contrast, the response letter refers to "neural-specialized" and "bona fide neural progenitors," and the manuscript describes transcription-factor$^+$/smedwi-1$^+$ cells as "newly specified neural progenitors" (lines 205-206).
Previous studies support enrichment of neural fate-associated factors in tgs-1$^+$ neoblasts, but do not directly establish a neural progenitor identity or developmental potential. Raz et al. showed that fate-associated neoblast states can retain broader potency. These findings argue for caution in assigning lineage identity from marker expression alone.
The EdU experiments show increased incorporation of neoblast-derived cells into neural tissues, but do not link those cells specifically to the tgs-1$^+$, pax6a$^+$, otxA$^+$, sim$^+$, or nkx2.1$^+$ populations quantified earlier. These are parallel observations rather than a demonstrated lineage sequence. Although direct lineage-tracing data are not available, the text should distinguish neural fate enrichment from demonstrated neural progenitor identity and should acknowledge that the source of the newly incorporated neurons was not directly established.
2. The marker analyses do not exclude an injury-induced state. The authors argue that increases in pax6a$^+$, otxA$^+$, sim$^+$, nkx2.1$^+$, and tgs-1$^+$ populations make such a state unlikely. However, an injury-induced state could involve coordinated expression of several neural-associated genes rather than isolated expression of one marker. The markers were also examined separately and were not shown to be co-expressed within the same cells. Similarly, the absence of early induction in bulk-expression data does not exclude a response in a smaller neoblast population. The data show amplification of several neural-associated neoblast populations, but do not distinguish stable progenitor populations from transient injury-associated states. The corresponding conclusions should be revised.
3. The scope of the proposed generic progenitor response should be clarified. The pharyngeal, muscle, and epidermal analyses provide useful evidence for bystander-like or spatially imprecise responses in several tissue contexts. However, these progenitor populations were examined using different injuries, anatomical regions, and time points. The experiments therefore do not test whether unrelated progenitor populations are coordinately amplified within the same regenerative context. The authors acknowledge in their response that not all specialized neoblast classes were examined and may not behave similarly. This qualification should also be clear in the manuscript and model. The manuscript and model should therefore avoid implying that the observed responses represent coordinated or equivalent amplification of all local progenitor classes.
4. The central conclusion remains broader than the data support. The experiments show that loss of a particular mature tissue is not strictly required for increased production or incorporation of several examined cell types. They do not establish a general lack of specificity in planarian progenitor responses or exclude tissue-specific feedback. Although the revised discussion acknowledges that generic and tissue-specific mechanisms may coexist, the title and several statements in the abstract and conclusion remain definitive. These sections should emphasize that generic amplification and bystander incorporation contribute to planarian regeneration and can coexist with tissue-specific regulatory mechanisms.

Reviewer 3

The Authors have significantly expanded the manuscript, addressing our and other Reviewer's requests and questions. We note that this expansion, requested by Reviewers, has further made the paper a laborious read, despite the efforts of the Authors to provide schematics. In our view, the manuscript is acceptable for publication, but we still invite the authors to simplify the text for a broader readership, if they can.

© 2026. Published by The Company of Biologists under the terms of the Creative Commons Attribution License (https://creativecommons.org/licenses/by/4.0/).

Second revision

Author response to reviewers' comments

Reviewer 1: The authors have satisfactorily addressed all the issues raised by the reviewer.

Reviewer 2: SUMMARY OF THE ADVANCE MADE IN THIS PAPER AND ITS POTENTIAL SIGNIFICANCE TO THE FIELD

The revised manuscript is strengthened by the new analyses of neural-associated neoblasts and progenitors of other lineages. Several issues of interpretation remain.

SUGGESTIONS TO AUTHORS:

1. The interpretation of tgs-1$^+$ neoblasts as neural progenitors remains overstated. The new data show that tgs-1$^+$/smedwi-1$^+$ cells increase after anterior-flank injury. This establishes amplification of a tgs-1- expressing neoblast population, but not its developmental output. The manuscript describes tgs-1$^+$ cells as "neural-fate-enriched neoblasts." This wording should be retained. By contrast, the response letter refers to "neural-specialized" and "bona fide neural progenitors," and the manuscript describes transcription-factor$^+$/smedwi-1$^+$ cells as "newly specified neural progenitors" (lines 205-206).

(Note that we added *tgs-1* to the revision at the request of reviewer 2 in round 1). In lines 205-210, we had "*transcription-factor+/smedwi-1+*" and "*tgs-1+/smedwi-1+*" semantically separated; we added "neural" and "neural-fate-enriched" to further clarify we mean to differentiate the *tgs-1*+ population throughout the paragraph. Additionally, we modified how we introduce *tgs-1*+ neoblasts in lines 194-202:

"In parallel, to interrogate progenitors we utilized RNA probes to neural transcription factor (TF)- encoding gene transcripts in smedwi-1+ cells: pax6a (dd_17726), expressed broadly in neural progenitors (Scimone et al., 2014), otxA (dd_14633), associated with cephalic ganglion neurons and photoreceptors (Lapan and Reddien, 2011; Scimone et al., 2014), nkx2.1 (dd_13898), expressed in brain neurons (Scimone et al., 2014), and sim (dd_17731), required for the regeneration of several neuron types (Cowles et al., 2013; Scimone et al., 2014). We also examined neural-fate-enriched tgs-1+ (dd_10988); smedwi-1+ neoblasts (King et al., 2024; Raz et al., 2021; Zeng et al., 2018)."

And in lines 209-210:

"Because smedwi-1 transcription ceases as neoblasts exit the cell cycle, neural transcription factor+; smedwi-1+ cells are newly produced. There was a significant increase in neural transcription factor+; smedwi-1+ and neural-fate-enriched tgs-1+; smedwi-1+ cells by 5 dpi following anterior-flank injury in all cases, with sim+, nkx2.1+, and otxA+ cells also showing a significant increase at 3 dpi (Fig. 2K-L). Whereas lineage-tracing is unavailable to link progenitors to mature cells, these observations further suggest that direct injury to the brain is not necessary for amplification of some neural progenitors. "

Previous studies support enrichment of neural fate-associated factors in tgs-1$^+$ neoblasts, but do not directly establish a neural progenitor identity or developmental potential. Raz et al. showed that fate- associated neoblast states can retain broader potency. These findings argue for caution in assigning lineage identity from marker expression alone.

The EdU experiments show increased incorporation of neoblast-derived cells into neural tissues, but do not link those cells specifically to the tgs-1$^+$, pax6a$^+$, otxA$^+$, sim$^+$, or nkx2.1$^+$ populations quantified earlier. These are parallel observations rather than a demonstrated lineage sequence.

© 2026. Published by The Company of Biologists under the terms of the Creative Commons Attribution License (https://creativecommons.org/licenses/by/4.0/).

Although direct lineage-tracing data are not available, the text should distinguish neural fate enrichment from demonstrated neural progenitor identity and should acknowledge that the source of the newly incorporated neurons was not directly established.

To aid in clarity, we changed the language in this section to make it clearer the analyses were done in parallel and that we do not intend to imply a connection between the interrogated progenitors and particular mature neurons:

Lines 192: *"To interrogate progenitors in parallel, we utilized RNA probes to neural (primarily transcription factor (TF)-encoding) gene transcripts in smedwi-1+ cells…"*

Lines 209-210: *"Whereas lineage-tracing is unavailable to link progenitors to mature cells, these observations further suggest that direct injury to the brain is not necessary for amplification of some neural progenitors."*

2. The marker analyses do not exclude an injury-induced state. The authors argue that increases in pax6a$^+$, otxA$^+$, sim$^+$, nkx2.1$^+$, and tgs-1$^+$ populations make such a state unlikely. However, an injury- induced state could involve coordinated expression of several neural-associated genes rather than isolated expression of one marker. The markers were also examined separately and were not shown to be co-expressed within the same cells. Similarly, the absence of early induction in bulk-expression data does not exclude a response in a smaller neoblast population. The data show amplification of several neural-associated neoblast populations, but do not distinguish stable progenitor populations from transient injury-associated states. The corresponding conclusions should be revised.

We now note (lines 201-204) that we cannot rule out that a small fraction of neoblasts might exhibit a neural-like injury-induced program. In lines 209-214 we emphasize lineage tracing is unavailable.

199-202: *"None of the neural TFs examined are wound-induced by RNA sequencing, but instead they increase in transcript level during the MTR of head regeneration, when head progenitors are amplified (Fig. S2E); although undetected, we cannot exclude a possible neural-like injury-induced program in a small neoblast subset."*

Lines 209-210: *"Whereas lineage-tracing is unavailable to link progenitors to mature cells, these observations further suggest that direct injury to the brain is not necessary for amplification of some neural progenitors."*

3. The scope of the proposed generic progenitor response should be clarified. The pharyngeal, muscle, and epidermal analyses provide useful evidence for bystander-like or spatially imprecise responses in several tissue contexts. However, these progenitor populations were examined using different injuries, anatomical regions, and time points. The experiments therefore do not test whether unrelated progenitor populations are coordinately amplified within the same regenerative context. The authors acknowledge in their response that not all specialized neoblast classes were examined and may not behave similarly. This qualification should also be clear in the manuscript and model. The manuscript and model should therefore avoid implying that the observed responses represent coordinated or equivalent amplification of all local progenitor classes.

We note that the words "coordinated" and "equivalent" are not used in the manuscript; indeed it is not our intent to claim something put in those terms. Injuries were selected to best test the hypothesis of specificity of production toward the injured tissue - for instance, the same injuries that are appropriate to interrogate the brain are not necessarily appropriate for the pharynx. As several planarian tissues show differing differentiation timelines, selecting identical time points across tissues when assessing incorporation into mature tissues is not always feasible or appropriate.

We amended language further (lines 440-442) to clarify that not all specialized neoblasts classes might behave the same: *"We also considered how new-cell incorporation after injury might differ in uniformly-distributed tissues, such as muscle, peripheral neurons, and epidermis; any injury removing body regions would infringe on these cells. This could, in*

© 2026. Published by The Company of Biologists under the terms of the Creative Commons Attribution License (https://creativecommons.org/licenses/by/4.0/).

principle, generate conditions for different evolved progenitor behavior compared to regionalized tissues, and it remains possible that not all progenitors—regional or uniform—will behave the same."

4. The central conclusion remains broader than the data support. The experiments show that loss of a particular mature tissue is not strictly required for increased production or incorporation of several examined cell types. They do not establish a general lack of specificity in planarian progenitor responses or exclude tissue-specific feedback. Although the revised discussion acknowledges that generic and tissue-specific mechanisms may coexist, the title and several statements in the abstract and conclusion remain definitive. These sections should emphasize that generic amplification and bystander incorporation contribute to planarian regeneration and can coexist with tissue-specific regulatory mechanisms.

We note that we stated in the original and revised versions of the manuscript that the data does not exclude tissue-specific feedback. That said, we modified the title to more clearly reflect conclusions, and edited wording in the discussion (lines 497-492) to even further emphasize that tissue-specific regulatory could contribute to regenerative outcomes and be consistent with the existence of the bystander effect. We amended language in the abstract (lines 14-16) similarly.

14-16: ***"We suggest that planarian regeneration specificity involves in large part a combination of ongoing cell turnover, wound-associated increased stem cell division, and spatially broad neoblast specification zones."***

497-492: ***"Based on the work presented here, contextualized within the field, we propose a general model contributes to planarian regeneration specificity: the coincidence of constitutive positional information with elevated proliferation results in progenitor amplification from broad specification zones to ensure that wounds generally receive the progenitors they need (Fig. 7B). However, the specificity of this mechanism is not precise - a bystander effect is seen where increased production of cell-types occurs even for uninjured tissues. Even after direct injury, incorporation is observed outside of the wound site. In axolotls, brain injury increased new neuron incorporation into a distal uninjured region (Amamoto et al., 2016) - raising the possibility that cell-type-production amplification outside of the wound, as described here, might occur broadly across regenerative contexts. We conclude that the identity of mature tissue itself is unlikely to be the sole determinant of specificity of progenitor responses to injury in planarian regeneration, with regeneration specificity instead being guided at least in part by the broad factors of positional information and stem cell proliferation, factors extrinsic to the tissue itself. "***

Reviewer 3: The Authors have significantly expanded the manuscript, addressing our and other Reviewer's requests and questions. We note that this expansion, requested by Reviewers, has further made the paper a laborious read, despite the efforts of the Authors to provide schematics. In our view, the manuscript is acceptable for publication, but we still invite the authors to simplify the text for a broader readership, if they can.

We endeavored to improve transitions between text sections to improve readability for a broad audience.

Third decision letter

MS ID#: dev.205573R2

MS TITLE: Planarian progenitor responses to injury are not specific to missing tissue identity

AUTHORS: Cecilia Pellegrini; Peter W Reddien

© 2026. Published by The Company of Biologists under the terms of the Creative Commons Attribution License (https://creativecommons.org/licenses/by/4.0/).

Dear Peter,

I am happy to tell you that your manuscript has been accepted for publication in Development, pending our standard publication integrity checks.

© 2026. Published by The Company of Biologists under the terms of the Creative Commons Attribution License (https://creativecommons.org/licenses/by/4.0/).
